# Osteological Variation among Extreme Morphological Forms in the Mexican Salamander Genus *Chiropterotriton* (Amphibia: Plethodontidae): Morphological Evolution And Homoplasy

**DOI:** 10.1371/journal.pone.0127248

**Published:** 2015-06-10

**Authors:** David M. Darda, David B. Wake

**Affiliations:** 1 Department of Biological Sciences, Central Washington University, Ellensburg, Washington, United States of America; 2 Department of Integrative Biology and Museum of Vertebrate Zoology, University of California, Berkeley, California, United States of America; Trier University, GERMANY

## Abstract

Osteological variation is recorded among and within four of the most distinctive species of the Mexican salamander genus *Chiropterotriton*. Analysis of the data is consistent with the monophyletic status of the genus and documents previously unrecorded intraspecific and interspecific variation. Most of the recorded variation involves qualitative and quantitative proportional differences, but four fixed differences constitute autapomorphic states that affirm and diagnose some species (*C*. *dimidiatus*, *C*. *magnipes*). Osteological variation in 15 characters is analyzed with respect to predictions generated from four hypotheses: 1) phylogeny, 2) adaptation to specific habitats (the four species include cave-dwelling, terrestrial, and arboreal forms), 3) size-free shape, and 4) size. High levels of intraspecific variation suggest that the characters studied are not subject to rigid functional constraints in salamanders, regardless of size. The pattern predicted by the hypothesis based on size differences seen among these four *Chiropterotriton* species matches most closely the observed pattern of relative skull robustness. Since size change and heterochrony are often associated in plethodontid evolution, it is likely that changes in developmental timing play a role in the morphological transitions among these morphologically diverse taxa. Webbed feet, miniaturization, body shape, and an unusual tarsal arrangement are morphologies exhibited in species of *Chiropterotrition* that are shown to be homoplastic with other clades of tropical plethodontids. Although extensive homoplasy in salamanders might be seen as a roadblock to unraveling phylogenetic hypotheses, the homologous developmental systems that appear to underlie such homoplasy may reveal common and consistent evolutionary processes at work.

## Introduction

Most salamanders are restricted to the temperate regions of the Northern Hemisphere, where all ten families occur. Only one family, the Plethodontidae, has successfully invaded and occupied tropical regions. This family contains approximately 446 species (66% of all salamanders), distributed mainly in the New World. Approximately 285 of these species, or 65%, all members of the tribe Bolitoglossini, occur in neotropical regions, from northern Mexico to the Amazon Basin of Peru, Bolivia, and Brazil [1–3].

Wake [4–6] and Wake and Lynch [2] have argued that the absence of an aquatic larval stage and the evolution of a highly sophisticated tongue projection feeding system [7, 8] in these neotropical plethodontids (tribe Bolitoglossini) may have facilitated such an invasion into the relatively densely crowded, predator-rich tropics. Whatever conditions enabled these salamanders to first occupy the Neotropics, the success of their adaptive radiation is undeniable, and the many species of this group occupy varied habitats and show a correlated diversity of form [5].

Such morphological diversity within a monophyletic lineage presents an opportunity to study the pattern of morphological evolution and the underlying processes responsible for evolutionary transitions. Such investigations have been successfully undertaken for portions of this lineage [1, 2, 9–16]. Nevertheless, understanding of the entire evolutionary pattern and process has been hindered by the rampant homoplasy in these neotropical plethodontids [1, 4, 6, 17, 18]. Morphological "syndromes", such as body and tail elongation, miniaturization, gigantism, increased relative limb length, and expansion of digital webbing, have evolved several times within the group. One is therefore faced with the question of how the diverse morphologies evolved, as well as, how such diversity has been "bounded" or constrained to produce the parallel syndromes seen in this group of salamanders. Wake and Elias [1] suggest that such parallelism may result from extreme specialization, and that there may be little opportunity for further morphological innovation in this highly derived lineage. Such precise adaptations of these animals to their environment may be viewed developmentally as a sequence of bifurcations between possible internal states that produce a finite set of morphological endpoints [17, 19, 20].

**Fig 1:**
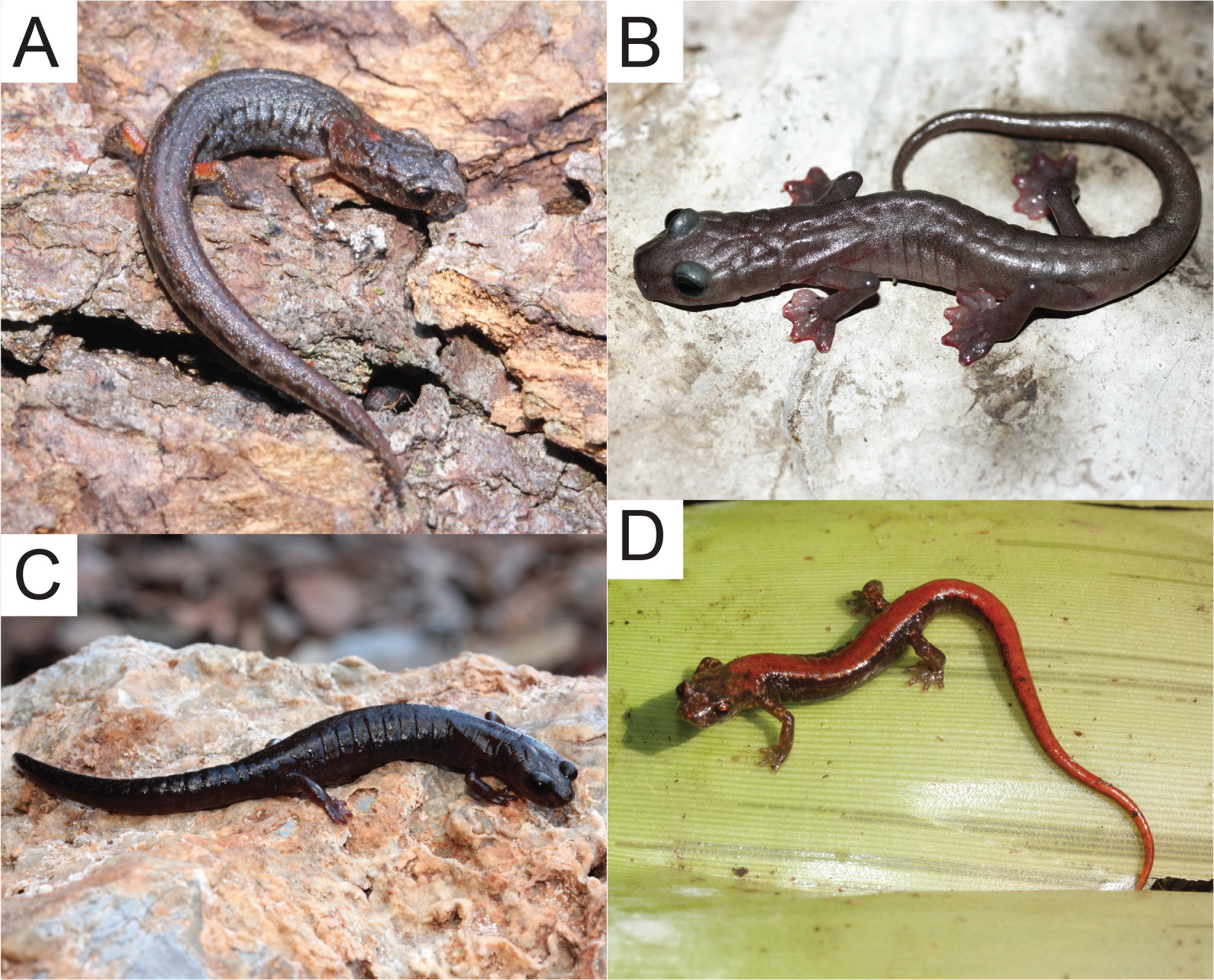
Photographs of the four species of *Chiropterotriton* examined in this study. (A) *C*. *dimidiatus* (adult SVL = approx. 25 mm); (B) *C*. *magnipes* (adult SVL = approx. 55 mm); (C) *C*. *priscus* (adult SVL = approx. 40 mm); (D) *C*. *lavae* (adult SVL = approx. 30 mm). Photos reprinted under a CC BY license, with permission from Sean Rovito.

The monophyletic genus *Chiropterotriton*, a member of this neotropical lineage of plethodontid salamanders, presents an intriguing example of such diversity and homoplasy. *Chiropterotriton* is one of the most morphologically diverse neotropical genera. The morphological extremes in *Chiropterotriton* are seen in three species *C*. *dimidiatus*, *C*. *magnipes*, and *C*. *priscus* (Fig 1, S1 Permission). *Chiropterotriton dimidiatus*, as the name implies, is diminutive (adult snout-vent length (SVL) = approx. 25 mm), with short limbs, little interdigital webbing, and a relatively short, thick-based, strongly tapering tail. At the other end of the spectrum, *C*. *magnipes* a cave-dwelling species, is not only impressive in its relatively large size (adult SVL = approx. 55 mm), but also in having long limbs, a long, slender tail, a wide, flattened head with bulging eyes, and, most distinctively, large, extensively webbed feet. *Chiropterotriton priscus* (adult SVL = approx. 40 mm) is an unusual *Chiropterotriton* in external shape. With its relatively stout body and short limbs, the species resembles species of other genera, and belie its relationship with the other, more gracile, members of *Chiropterotriton*, which form a rather smooth continuum between *C*. *dimidiatus* and *C*. *magnipes*, with varying degrees of robustness, limb length, and general body size. Within this continuum is the fourth species used in this study, *Chiropterotriton lavae* (adult SVL = approx. 30 mm), an arboreal specialist that lives in bromeliads and has a morphotype shared by several other Chiropterotriton species (collectively known as the *chiropterus* group) (Fig 1).

These morphological extremes are of further interest because each represents at least one morphological "syndrome" seen in other neotropical genera. The four species can be viewed as ecomorphs, associated with distinctive ways of life. The miniaturized *C*. *dimidiatus* superficially resembles largely terrestrial members of the Mexican genera *Parvimolge* and *Thorius*. Some *Thorius* represent the smallest terrestrial vertebrates [13, 15, 16, 20]. *Chiropterotriton magnipes* resembles (but see [21]) forms with extensive digital webbing such as many species of *Bolitoglossa* [10], as well as large, long-legged forms such as *Nyctanolis* [22]. *Chiropterotriton priscus*, the most atypically shaped member of the genus, appears to express a generalized body form much like that seen in some members of the genus *Pseudoeurycea* [2, 23].

Thus, we see in *Chiropterotriton* a microcosm of the pattern of morphological diversity and homoplasy found in the entire neotropical lineage. Any understanding of morphological evolution gained in this well-defined genus should lead to greater understanding of the mechanisms working to mold the extensive morphological radiation of neotropical bolitoglossine salamanders.

Here, the four species are compared with respect to osteology, with an emphasis on the bones of the skull. Quantitative comparisons are made when appropriate, and although little postcranial anatomy is considered here, the hands and feet are examined in some detail. Appropriate comparisons are also made to other bolitoglossines.

## Materials and Methods

**Fig 2:**
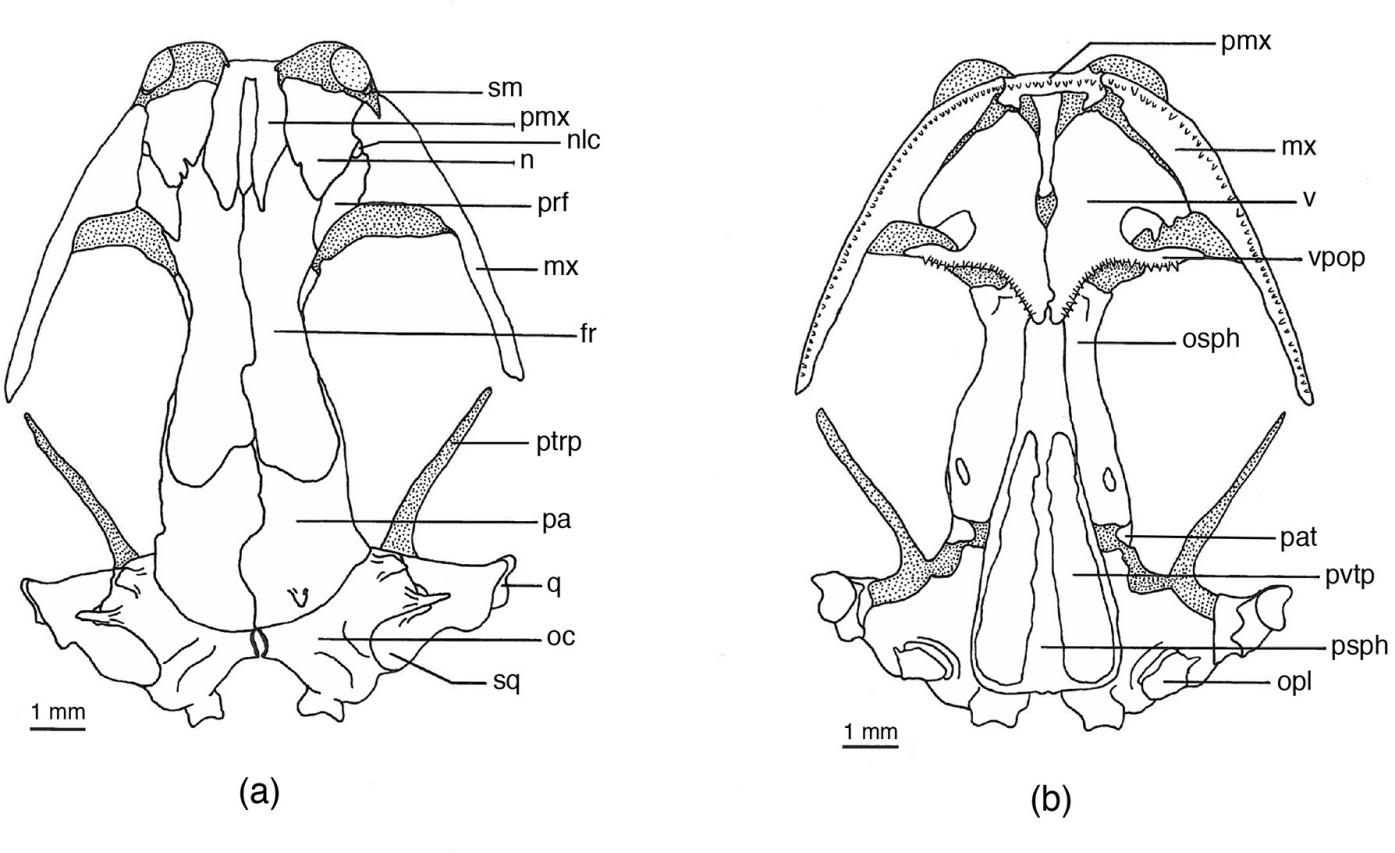
*Chiropterotriton magnipes* skull. Drawings of dorsal (a) and ventral (b) aspects of the skull of *Chiropterotriton magnipes* (MVZ 129021). Stippled areas represent cartilage. Posterior patch of vomerine teeth is outlined. Abbreviations: fr = frontal, mx = maxilla, n = nasal, nlc = nasolacrimal canal, oc = otic capsule, opl = opercular plate, osph = orbitosphenoid, pa = parietal, pat = parietal tab, pmx = premaxilla, prf = prefrontal, psph = parasphenoid, ptrp = pterygoid process, q = quadrate, sm = septomaxilla, sq = squamosal, v = vomer, vpop = vomer preorbital process, pvtp = posterior vomerine tooth patch.

**Fig 3:**
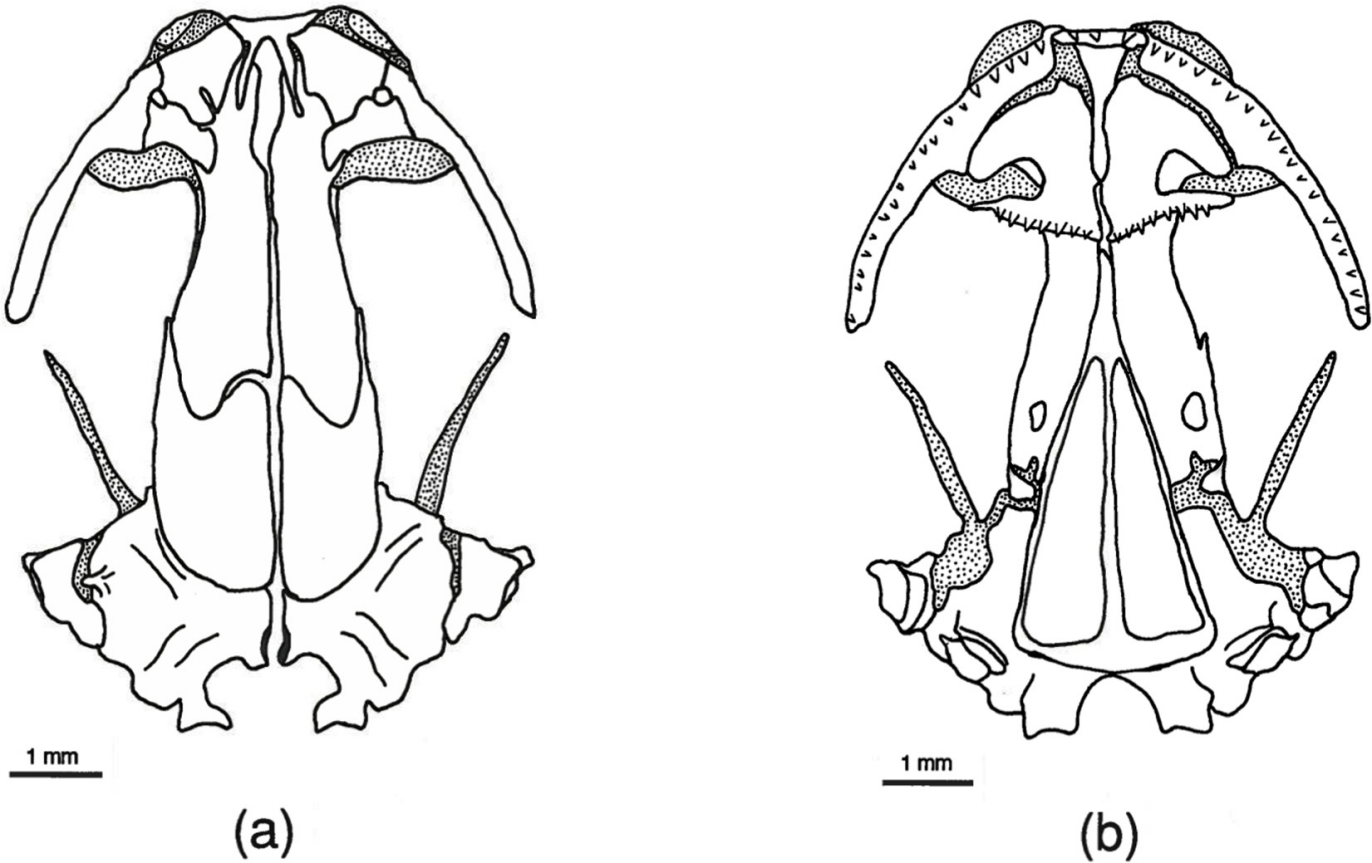
*Chiropterotriton priscus* skull. Drawings of dorsal (a) and ventral (b) aspects of the skull of *Chiropterotriton priscus* (MVZ 163887). Stippled areas represent cartilage. Elements as labeled in Fig 1.

**Fig 4:**
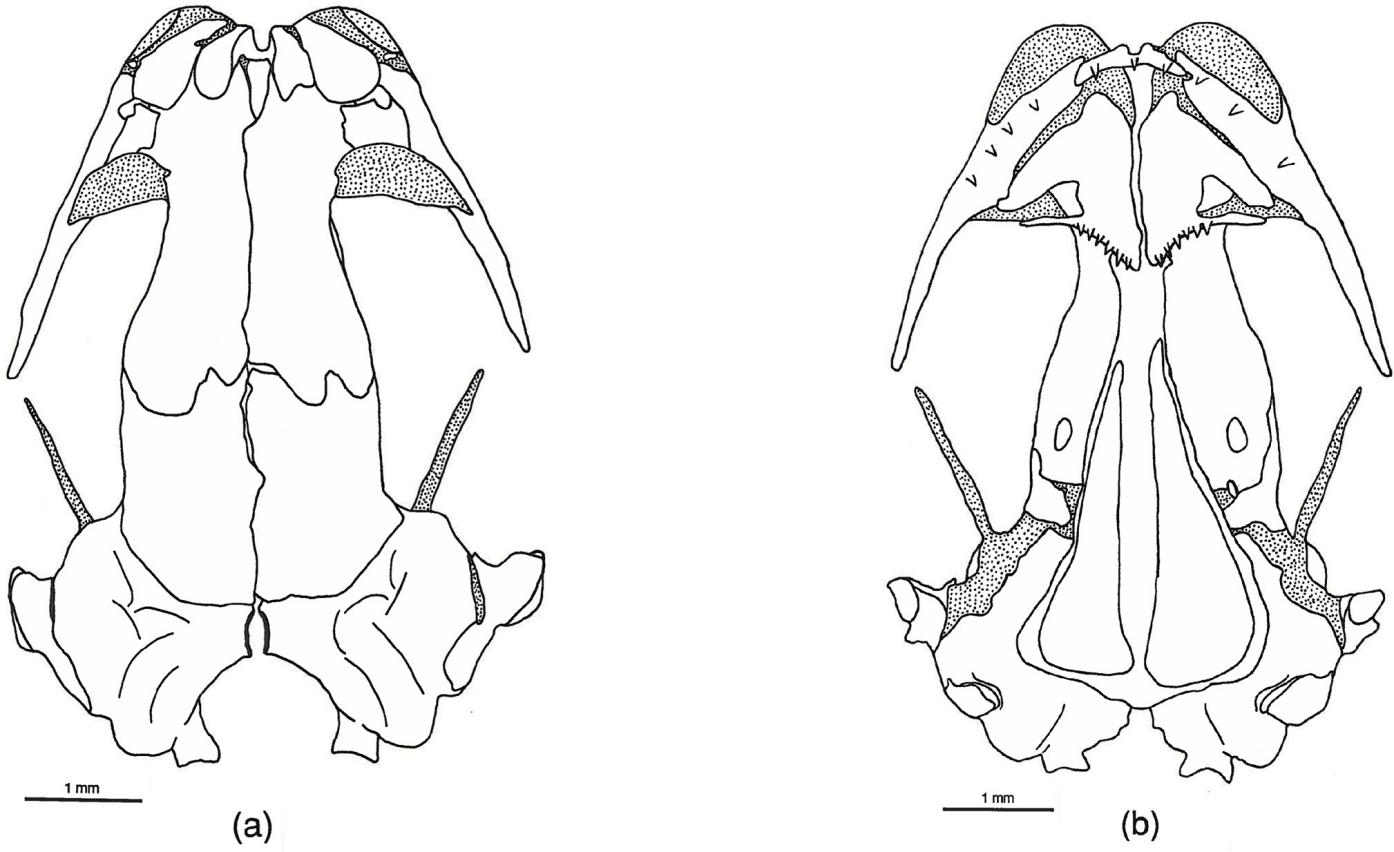
*Chiropterotriton lavae* skull. Drawings of dorsal (a) and ventral (b) aspects of the skull of *Chiropterotriton lavae* (MVZ 171898). Stippled areas represent cartilage. Elements as labeled in Fig 1.

**Fig 5:**
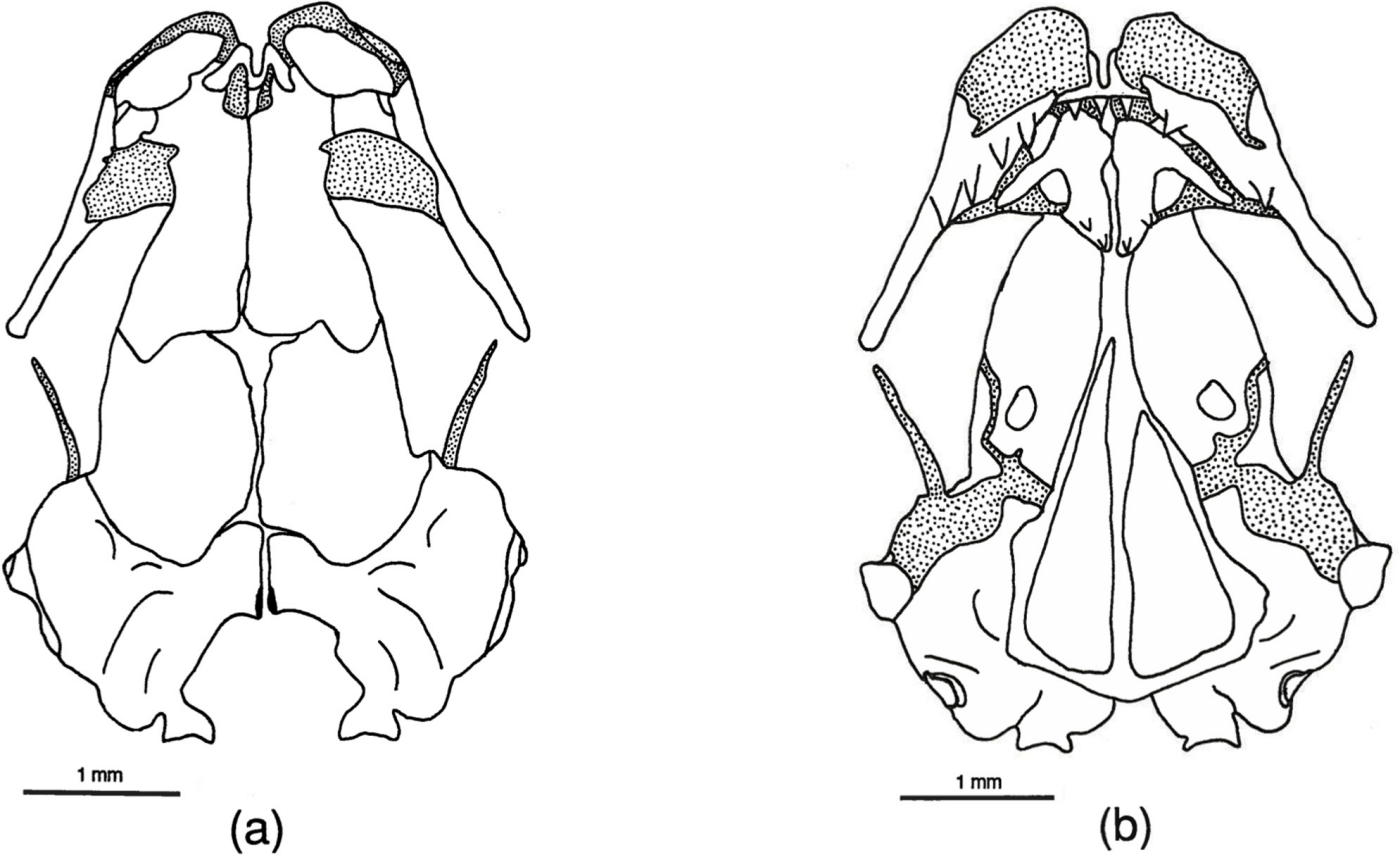
*Chiropterotriton dimidiatus* skull. Drawings of dorsal (a) and ventral (b) aspects of the skull of *Chiropterotriton dimidiatus* (MVZ 103967). Stippled areas represent cartilage. Elements as labeled in Fig 1.

**Fig 6:**
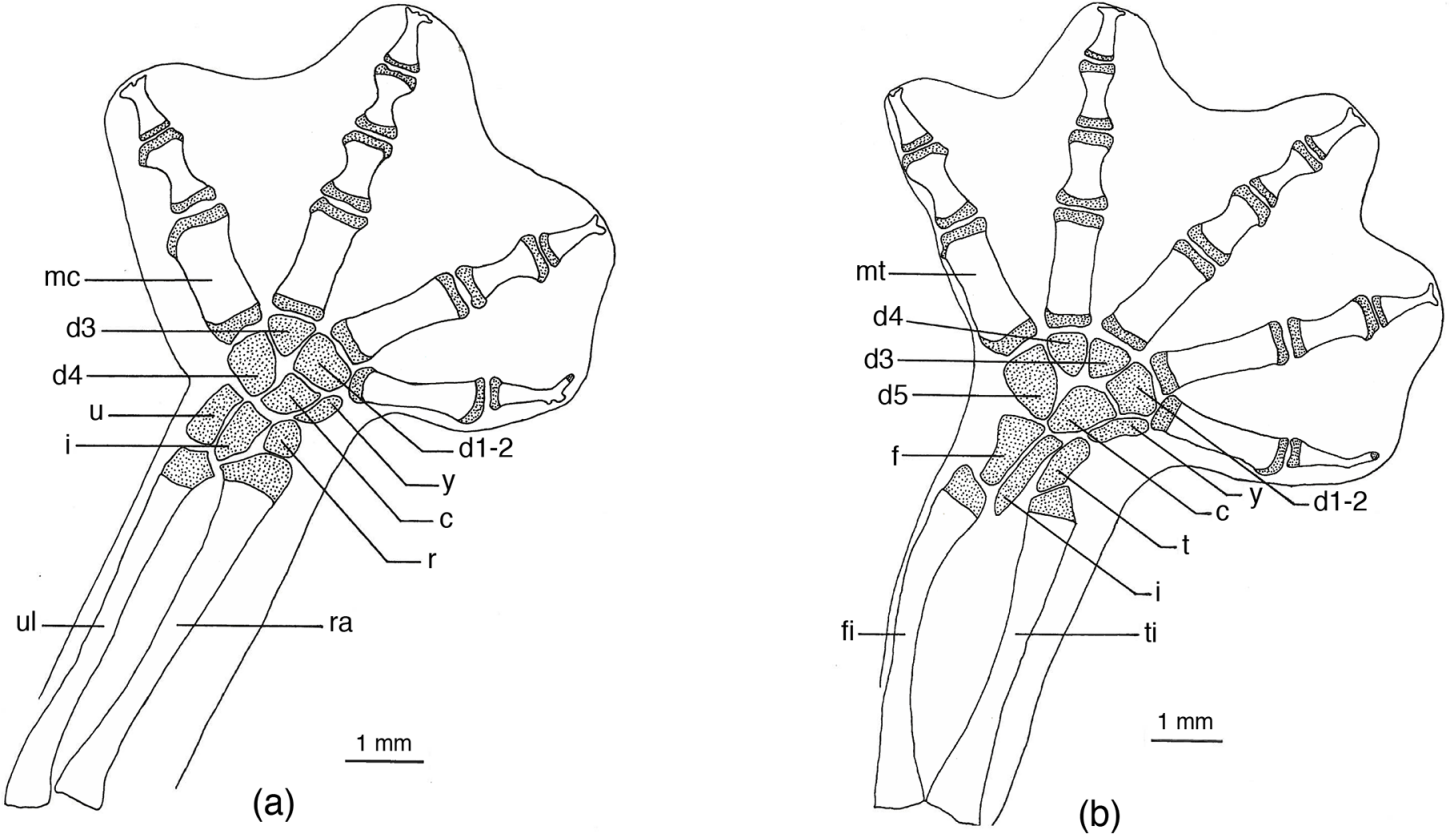
*Chiropterotriton magnipes* feet. Drawings of front, left (a) and rear, left (b) feet of *Chiropterotriton magnipes* (MVZ 129013), showing skeletal elements and the extent of interdigital webbing. Dorsal view; stippled areas represent cartilage. Abbreviations: c = centrale, d = distal carpals and tarsals, f = fibulare, fi = fibula, i = intermedium, mc = metacarplas, mt = metatarsals, r = radiale, ra = radius, t = tibiale, ti = tibia, u = ulnare, ul = ulna, y = element y.

**Fig 7:**
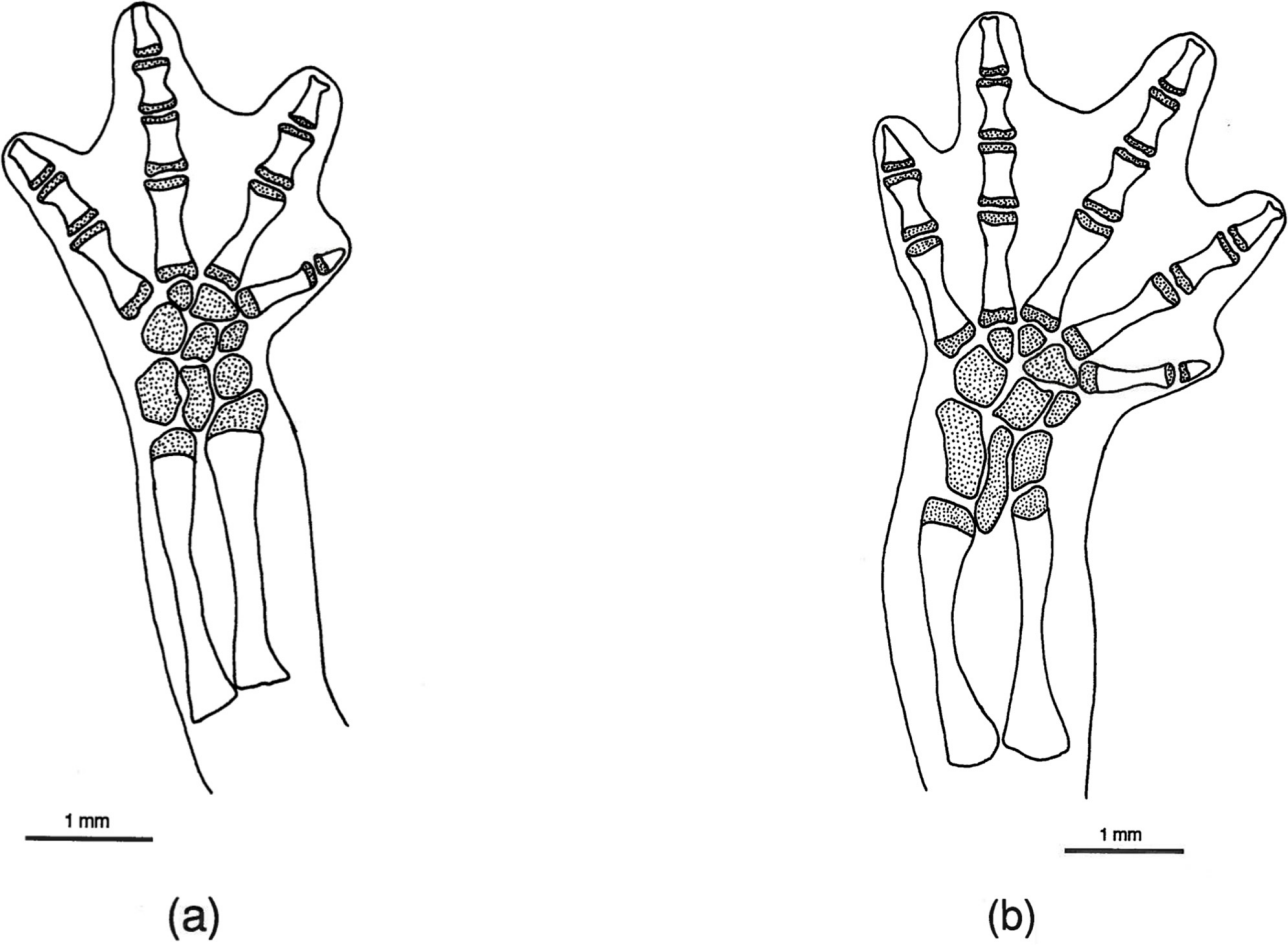
*Chiropterotriton priscus* feet. Drawings of front, left (a) and rear, left (b) feet of *Chiropterotriton priscus* (MVZ 192794), showing skeletal elements and the extent of interdigital webbing. Dorsal view; stippled areas represent cartilage. Elements as labeled in Fig 5.

**Fig 8:**
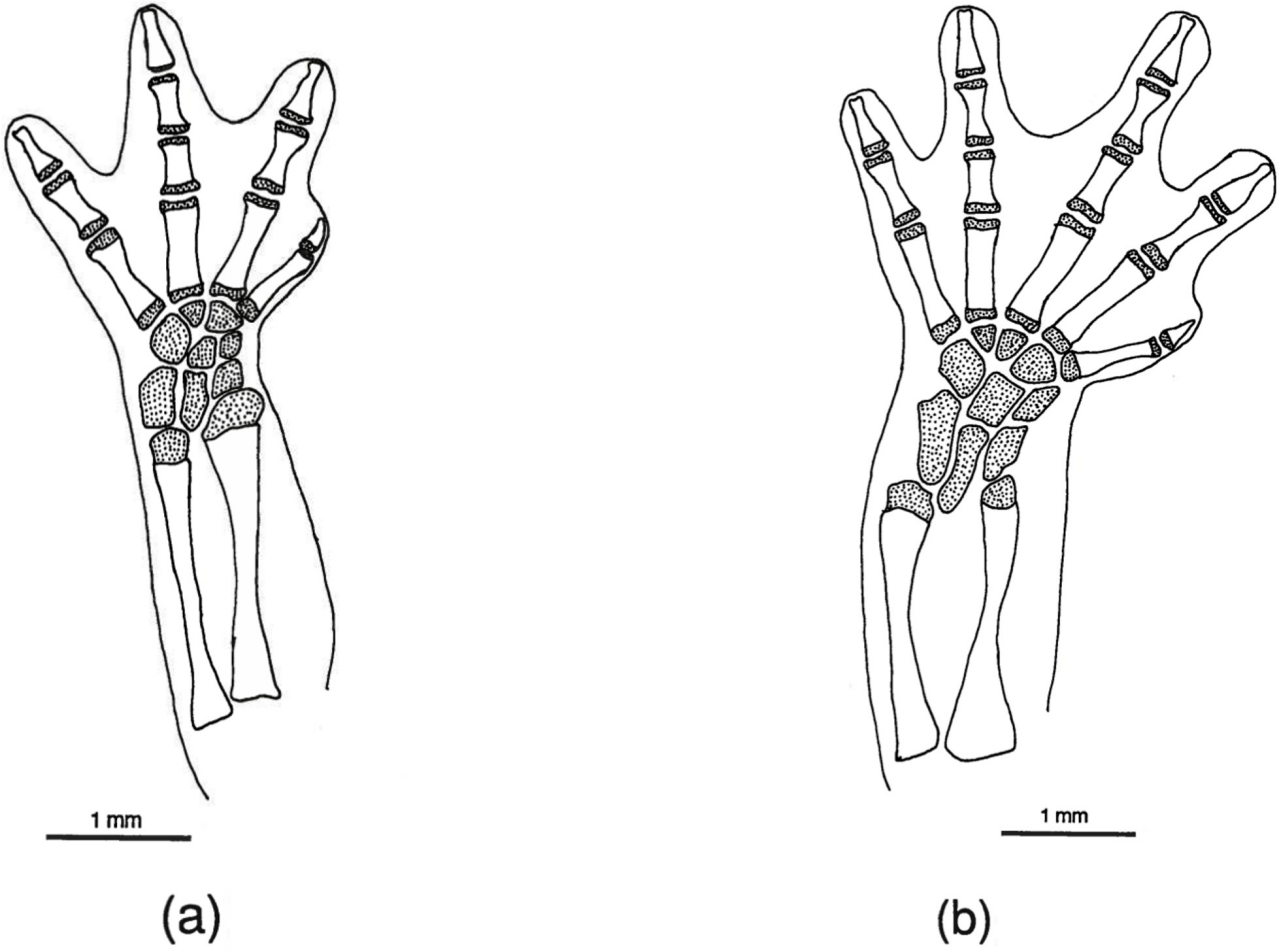
*Chiropterotriton lavae* feet. Drawings of front, left (a) and rear, left (b) feet of *Chiropterotriton lavae* (MVZ 171898), showing skeletal elements and the extent of interdigital webbing. Dorsal view; stippled areas represent cartilage. Elements as labeled in Fig 5.

**Fig 9:**
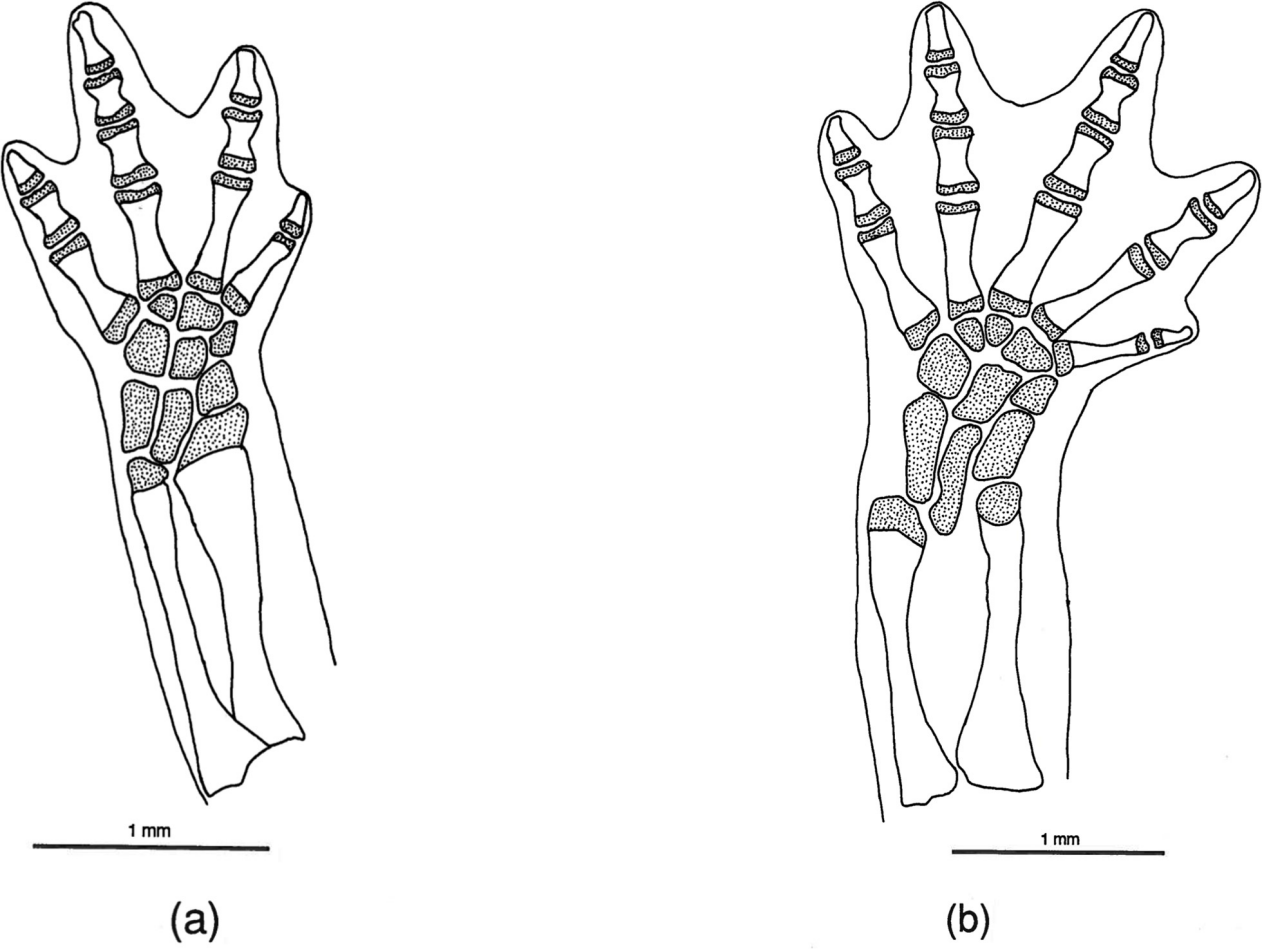
*Chiropterotriton dimidiatus* feet. Drawings of front, left (a) and rear, left (b) feet of *Chiropterotriton dimidiatus* (MVZ 185965), showing skeletal elements and the extent of interdigital webbing. Dorsal view; stippled areas represent cartilage. Elements as labeled in Fig 5.

Osteological variation was examined in four species of *Chiropterotriton* [mean SVL (mm) for males and females, respectively]: *C*. *dimidiatus* (25.8, 27.4); *C*. *lavae* (30.4, 32.0); *C*. *priscus* (41.2, 44.4); and *C*. *magnipes* (53.8, 55.8). The sample for each species consisted of five adult males and five adult females collected at a single locality or, in the case of *C*. *magnipes*, two localities in close proximity. All *Chiropterotriton* specimens as well as other comparative material are a part of the permanent collections of the Museum of Vertebrate Zoology (S1 Appendix). Specimens were cleared and differentially stained for bone and cartilage using an alizarin red—alcian blue procedure [24]. Drawings of skulls (Figs 2–5) were produced using a camera lucida on a Wild binocular microscope. Drawings of feet (Figs 6–9) were made by projecting and tracing an image produced by a Bausch and Lomb projecting microscope.

In order to examine the osteological variation among these four species, all specimens were scored for the following characters (Tables 1–4):

Anterior Skull

1. Septomaxilla development: (a) absent (b) present
2. Nasal-premaxilla articulation: (a) separate (b) abut (c) overlap (d) fused
3. Nasal-maxilla articulation: (a) separate (b) abut (c) overlap (d) fused
4. Nasal-prefrontal articulation: (a) separate (b) abut (c) overlap (d) fused
5. Nasal-frontal articulation: (a) separate (b) overlap

Posterior Skull

**Fig 10:**
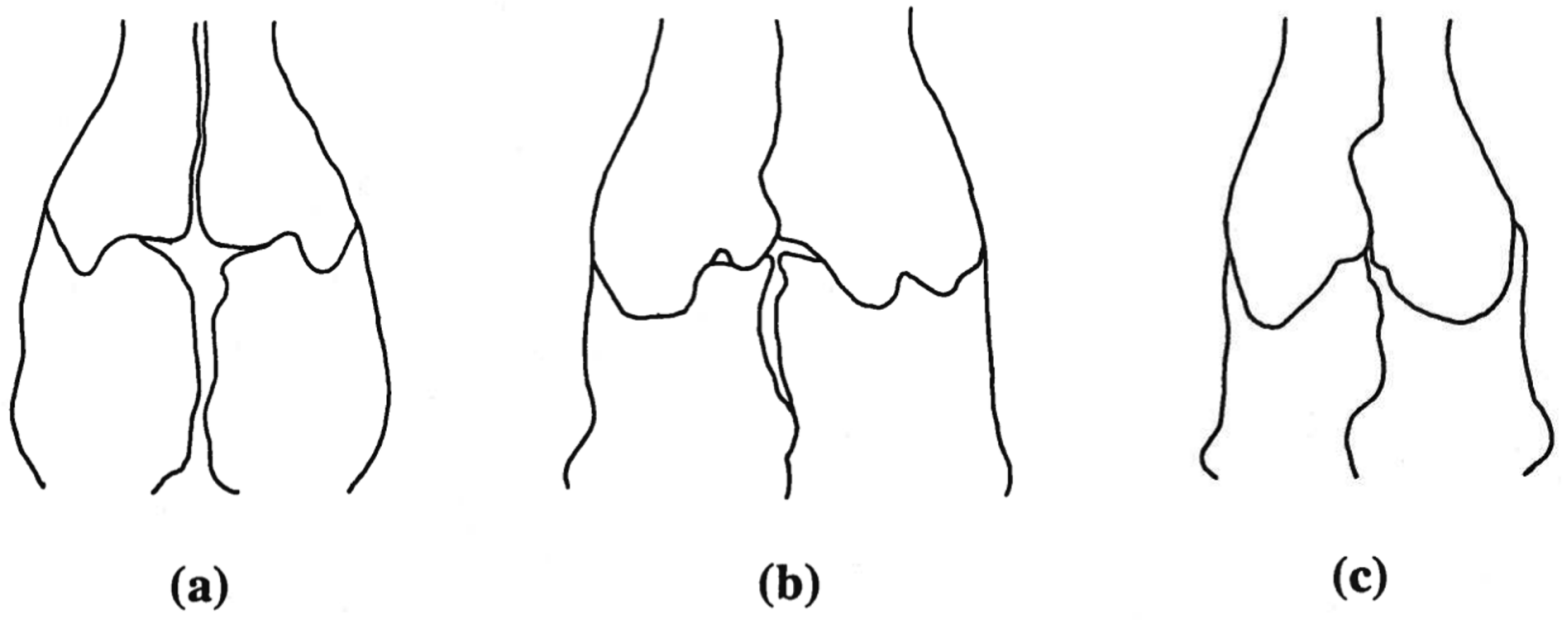
Frontoparietal fontanelle development. Three degrees of development of the paired frontal and parietal bones which result in the frontoparietal fontanelle being extensive (a), reduced (b), or absent (c) in *Chiropterotrition*. Osteological character 8.

6 Frontal-frontal articulation: (a) separate (b) abut (c) overlap (d) interdigitate
7 Parietal-parietal articulation: (a) separate (b) abut (c) overlap (d) interdigitate
8 Frontoparietal fontanelle: (a) extensive (b) reduced (c) absent (Fig 10)
9 Parietal process development: (a) absent (b) present
10 Otic process development: (a) absent (b) one process present (c) two processes present
11 Squamosal process: (a) absent (b) present

Ventral Skull

12 Vomer preorbital process development: (a) absent (b) present but reduced (c) present
13 Orbitosphenoid-frontal-parietal articulation: (a) separate (b) solid articulation
14 Premaxilla functional tooth number (i.e. those teeth ankylosed to the bone)
15 Maxilla functional tooth number (i.e. those teeth ankylosed to the bone)

Post-cranial

**Fig 11:**
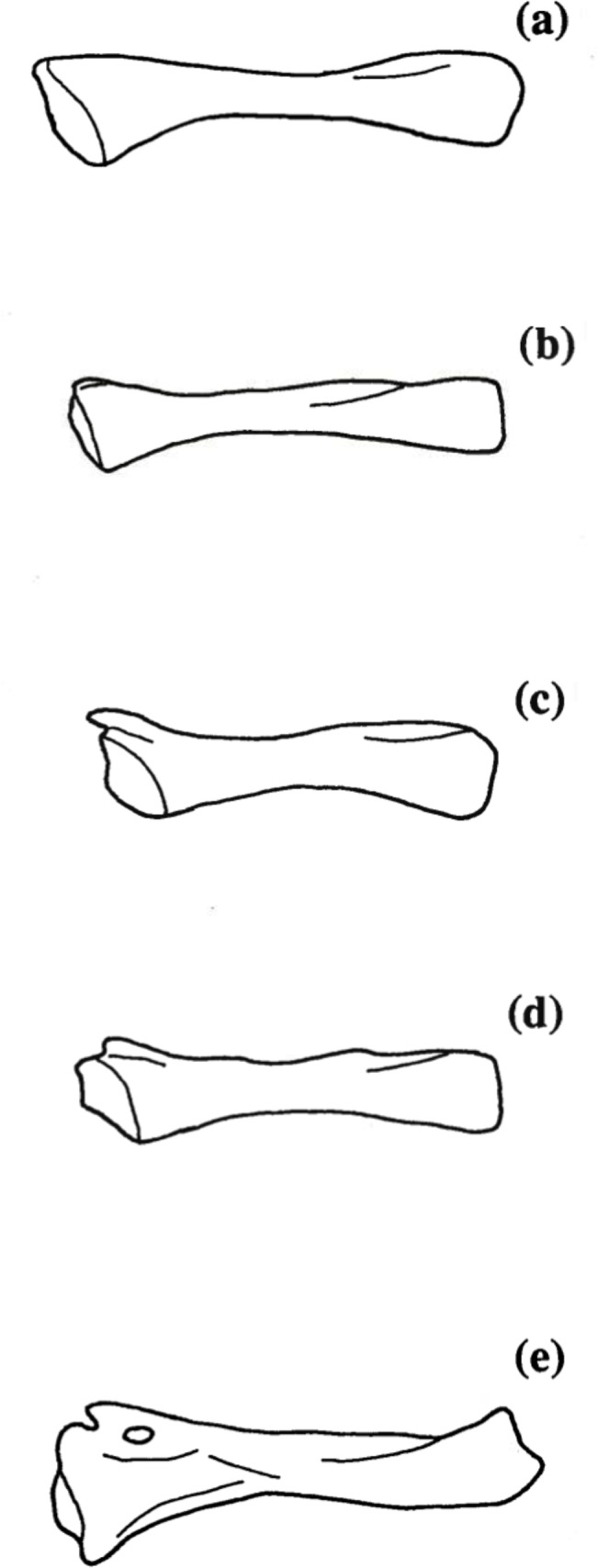
Tibial spur development. Five states of tibial spur development in *Chiropterotriton*: (a) absent, tibia smooth; (b) absent, tibial ridge; (c) present, detached; (d) present, attached; (e) present, attached with foramen. Osteological character 16.

16 Tibial spur development: (a) absent, tibia smooth (b) absent, tibial ridge(c) present, detached (d) present, attached (e) present, attached with foramen (Fig 11)
17 Carpal arrangement: (a) normal (c) anomalous
18 Tarsal arrangement: (a) normal (b) anomalous

**Table 1:** Frequency (%) of osteological character variation in the anterior skull.

Species	Character															
	1. Septomaxilla development		2. Nasal-premaxilla articulation				3. Nasal-maxilla articulation				4. Nasal-prefrontal articulation				5. Nasal-frontal articulation	
	absent	present	separate	abut	overlap	fused	separate	abut	overlap	fused	separate	abut	overlap	fused	separate	overlap
*C. magnipes*	10	90	40	5	55	-	-	-	100	-	10	5	85	-	-	100
*C. priscus*	5	95	100	-	-	-	-	5	95	-	20	15	65	-	-	100
*C. lavae*	10	90	80	20	-	-	10	10	70	10	85	10	-	5	-	100
*C. dimidiatus*	65	35	90	5	-	5	50	25	25	-	50	25	25	-	10	90

Frequencies calculated by considering right and left sides separately. Ten specimens of each species were examined.

**Table 2:** Frequency (%) of osteological character variation in the posterior skull.

Species	Character																	
	6. Frontal-frontal articulation				7. Parietal-parietal articulation				8. Frontoparietal fontanelle			9. Parietal process development		10. Otic process development			11. Squamosal process	
	separate	abut	overlap	interdigitate	separate	abut	overlap	interdigitate	extensive	reduced	absent	absent	present	absent	one process	two processes	absent	present
*C. magnipes*	-	-	60	40	-	-	30	70-	-	30	70	65	35	-	-	100	65	35
*C. priscus*	70	30	-	-	50	50	-	-	30	70	-	100	-	35	65	-	95	5
*C. lavae*	-	-	100	-	10	20	70	-	-	100	-	100	-	70	30	-	100	-
*C. dimidiatus*	30	40	30	-	100	-	-	-	100	-	-	100	-	90	10	-	100	-

Frequencies calculated by considering right and left sides separately. Ten specimens of each species were examined.

**Table 3:** Osteological character variation in the ventral skull.

Species	Character								
	12. Vomer preorbital process development*			13. Orbitosphenoid-frontal-parietal articulation*		14. Premaxilla tooth numbers**		15. Maxilla tooth numbers**	
	absent	present but reduced	present	solid articulation	separate	males	females	males	females
*C. magnipes*	-	-	100	100	-	12 (9–14)	15 (13–18)	70 (52–81)	80 (64–86)
*C. priscus*	-	-	100	100	-	3 (2–4)	4 (4–4)	24 (22–29)	35 (28–45)
*C. lavae*	-	30	70	100	-	4 (2–6)	7 (5–9)	12 (6–29)	34 (33–36)
*C. dimidiatus*	100	-	-	-	100	2 (2–3)	8 (3–6)	8 (5–9)	26 (20–37)

* Frequencies—calculated by considering right and left sides separately. Ten specimens of each species were examined.

** Tooth counts—number of functional teeth on the single, unpaired premaxilla and both right and left maxillae. Figures represent means, followed by the range in parentheses. Five males and five females of each species were examined.

**Table 4:** Frequency (%) of post-cranial osteological character variation.

Species	Character								
	16. Tibial spur development					17. Carpal arrangement		18. Tarsal arrangement	
	absent, tibia smooth	absent, tibial ridge	present, detatched	present, attached	present, attached with foramen	normal	anomalous	normal	anomalous
*C. magnipes*	40	60	-	-	-	100	-	100	-
*C. priscus*	-	-	-	5	95	95	5	95	5
*C. lavae*	-	-	70	30	-	100	-	100	-
*C. dimidiatus*	-	-	100	-	-	90	10	95	5

Frequencies calculated by considering right and left sides separately. Ten specimens of each species were examined.

Most of these character states represent discrete developmental stages, easily identifiable from specimen to specimen. Several characters (8, 12, 14, 15), however, are continuous, with the states representing easily identifiable, if somewhat subjective, reference points. States for characters 1–13 are arranged in ontogenetic sequence based on previous studies of plethodontid salamander skull development [11, 16, 25]. For paired characters (1–5, 9–11, 16–18), each side was scored independently. Frequency of right-left asymmetry was calculated.

## Results

### Skull Osteology (Figs 2–5)

#### General observations

The skulls of the four species of *Chiropterotriton* show a consistently high level of osteological development. This is consistent with previous observations [1,4] and can be seen in both the number of elements present and the degree of development of these elements. In each case, all bones one would expect to find in a plethodontid salamander skull are present, and each element is well developed.

**Fig 12:**
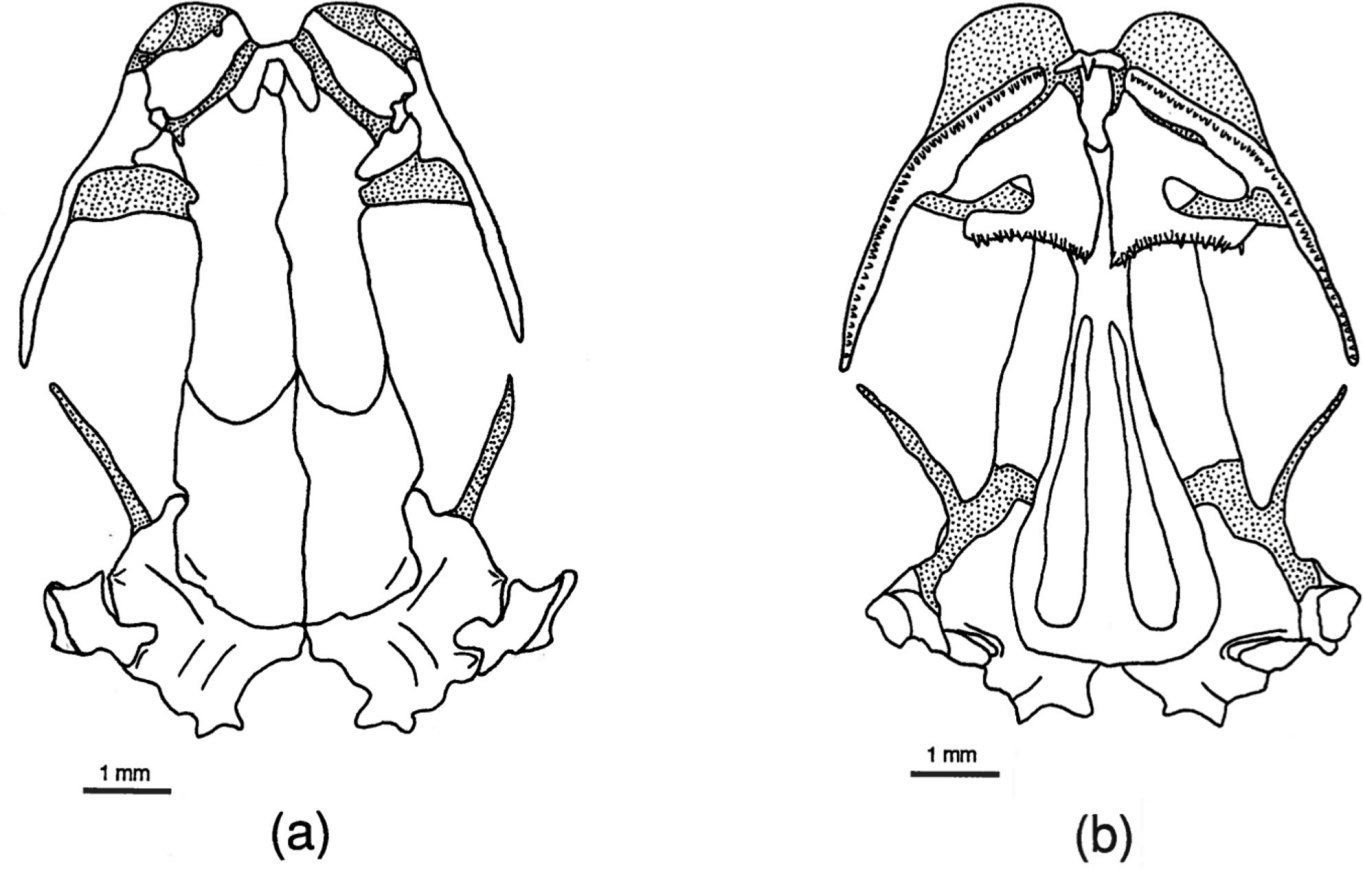
*Pseudoeurycea leprosa* skull. Drawings of dorsal (a) and ventral (b) aspects of the skull of *Pseudoeurycea leprosa* (MVZ 147092). Stippled areas represent cartilage. Elements as labeled in Fig 1.

The overall osteological pattern seen in these animals is suggestive of the skull of some members of the genus *Pseudoeurycea*, which, except for the usual lack of septomaxillary bones, exhibit one of the most generalized skull morphologies of the bolitoglossines (Fig 12) [4]. This level of generalization does not necessarily indicate that either of these genera is ancestral within the bolitoglossines, but suggests the likely ancestral skull morphology of this clade.

**Fig 13:**
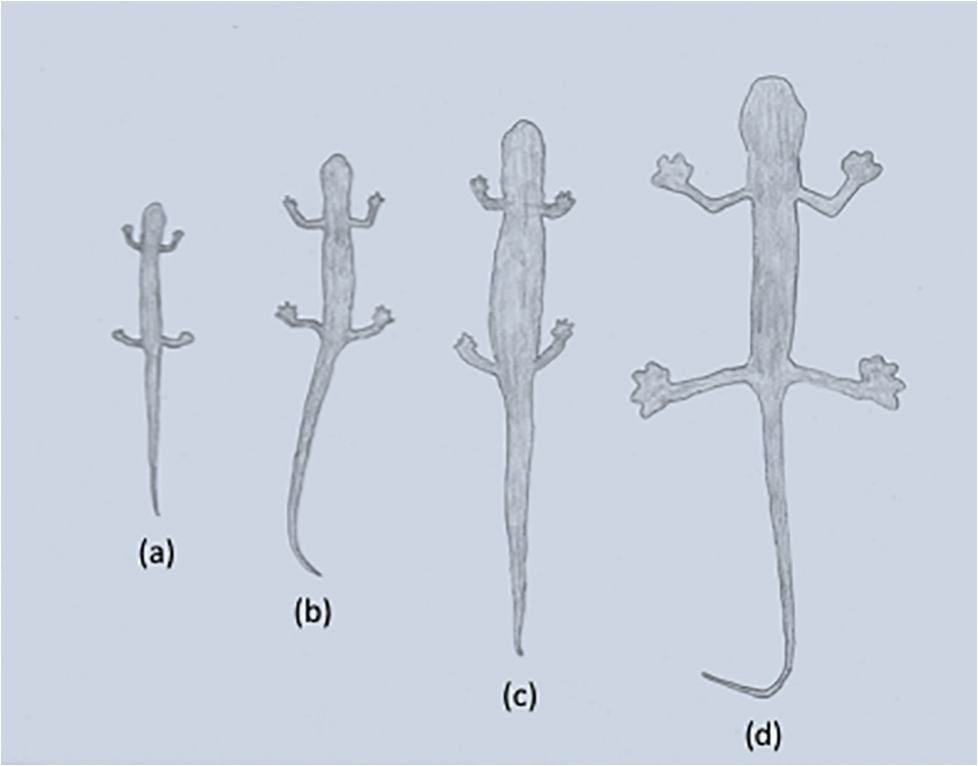
Relative size differences. Silhouette drawings illustrating the size differences among the four species of *Chiropterotriton* examined in this study. Rendered from a single photograph of the dorsal view of adult preserved specimens. (a) *C*. *dimidiatus* (MVZ 114511; SVL = 25 mm, total length = 50 mm); (b) *C*. *lavae* (MVZ 171876; SVL = 34 mm, total length = 69 mm); (c) *C*. *priscus* (MVZ 138885; SVL = 46 mm, total length = 86 mm); (d) *C*. *magnipes* (MVZ 129016; SVL = 52 mm, total length = 108 mm).

Osteology is generally consistent on a gross level across all four species, despite the great differences in overall size (Fig 13). The diminutive *C*. *dimidiatus* shows the same relatively high degree of ossification as the other species. Other diminutive bolitoglossines have been shown to have varying degrees of reduction in some anterior dermal bones [1, 4]. *Parvimolge townsendi*, like *C*. *dimidiatus*, has a relatively complete, compact skull [4]. Hanken [16] reported that in *Thorius*, the nasal, prefrontal, and septomaxillary bones were often either reduced to mere slivers or completely missing, leaving the nasal region of the skull almost entirely cartilaginous (although see [26]). Alberch [11] reported similar findings for small species of *Bolitoglossa*. In *C*. *dimidiatus*, the nasals and prefrontals are well developed and overlie a good proportion of the dorsal, anterior region of the cartilaginous olfactory capsule.

Because of the general similarity seen across these four taxa, the osteological characterizations presented here focus on more subtle aspects of skull proportion and development, most of which are recorded in Tables 1–3.

#### Anterior skull

The anterior portion of the skull is well developed in all four species. Of chief interest is the consistent presence of well-developed septomaxillary bones (Tables 1–4). These small, paired elements form at the posterolateral margin of the external nares, embedded in the cartilage of the olfactory capsule (Fig 2). They are primitively present in plethodontids but are greatly reduced or absent in many bolitoglossines [1, 4]. Among the bolitoglossines, septomaxillaries are best developed in *Chiropterotriton* [4, 23]. Of the specimens of *C*. *magnipes*, *C*. *priscus*, and *C*. *lavae* examined here, only a few lack these bones on one or both sides. Septomaxillae were present in *C*. *dimidiatus* only 35% of the time; they are often reduced in size and asymmetric in a given skull (Table 5). A single premaxilla is present in all *Chiropterotriton* and is typical of all neotropical plethodontids with the lone exception of *Nyctanolis* [1, 4]. In all specimens examined here, the frontal processes of the premaxillae arise separately and remain separate along their entire length.

**Table 5:** Frequency (%) of right-left asymmetry in paired osteological characters.

Species	Character								
	1. Septomaxilla development	2. Nasal-premaxilla articulation	3. Nasal-maxilla articulation	4. Nasal-prefrontal articulation	5. Nasal-frontal articulation	9. Parietal process development	10. Otic process development	11. Squamosal process	16. Tibial spur development
*C. magnipes*	20	10	-	20	-	30	-	30	40
*C. priscus*	10	-	10	30	-	-	30	10	10
*C. lavae*	-	20	40	30	-	-	-	-	-
*C. dimidiatus*	30	20	60	40	20	-	20	-	-

Ten specimens of each species were examined.

The nasal bones are broad and well developed in each of the four species examined. The shape of this element is variable among specimens of the same species, as well as between species. It can be quadrangular, triangular, or ovoid in shape, and is best described in its relation to surrounding bones (Table 1). The nasal is mostly separate from the premaxilla except in *C*. *magnipes*, where it overlaps the frontal process 55% of the time. Nasal articulation with the *pars facialis* of the maxilla is limited in its extent in all specimens, but at least some overlap is seen in all specimens of *C*. *magnipes* and most specimens of *C*. *priscus* and *C*. *lavae*. *C*. *dimidiatus* shows less articulation, with these elements separate in 50% of the cases. Nasal-prefrontal articulation is variable, with a high degree of overlap seen in *C*. *magnipes* and *C*. *priscus* and very little articulation of any kind in *C*. *lavae*. *C*. *dimidiatus* shows articulation of these elements in about 50% of the cases. The nasal and frontal bones overlap in all specimens of *C*. *magnipes*, *C*. *priscus*, and *C*. *lavae*, and in all but two *C*. *dimidiatus*, which show articulation on one side but not the other. Right-left asymmetry in all nasal articulations is most common in *C*. *dimidiatus* (Table 5).

Prefrontals are well developed in all four species. The anterior margins of these paired bones, in conjunction with dorsal maxillary and posterolateral nasal concavities, forms the foramen through which the nasolacrimal duct passes. In *C*. *magnipes*, *C*. *priscus*, and *C*. *lavae* each of these three bones contributes approximately equally to the formation of this foramen. In *C*. *dimidiatus*, however, the prefrontal envelops more of the opening and forms most of the medial and lateral margins, as well as the posterior margin.

Maxillae are relatively long, terminating at about the posterior extent of the eyes in all four species. The joints formed between the maxillae and premaxilla appear relatively strong in *C*. *priscus*, *C*. *lavae*, and *C*. *dimidiatus*. Each of these species has a flange-like extension of the maxilla that contacts and sometimes overlaps the *pars dentalis* of the premaxilla (Figs 3–6). This flange is less developed in *C*. *magnipes* and direct bone-to-bone articulation is less common than in the other species.

#### Dorsal skull

In dorsal aspect, the skull is dominated by the large, paired frontals and parietals. In most respects these bones in *Chiropterotriton* are typical of bolitoglossines [4], but variation within and among species is seen in the extent of articulation between these elements and between the right and left pairs of each set of bones. For instance, the two frontal bones in *C*. *priscus* do not contact each other at the midline in most specimens (70%), whereas all *C*. *lavae* show at least some overlap between these bones (Table 2). *Chiropterotriton dimidiatus* is variable in this respect, with approximately equal numbers showing the three categories: complete separation, abutment, and overlap. *Chiropterotriton magnipes* appears to take this articulation one step further. The two frontals overlap, but in an interdigitating manner: anteriorly the right element might overlap the left, then proceeding posteriorly, the left then overlaps the right, with this alternating pattern of overlap continuing along the midline articulation. No sagittal ridge, characteristic of mature *Aneides lugubris* with tightly articulating frontals [4, 25], was observed This interdigitation is seen in 40% of the *C*. *magnipes* examined, with the remaining animals showing simple overlap.

The parietal pairs show similar variation in articulation (Table 2). These elements are separate from each other in all *C*. *dimidiatus* examined and one half of the *C*. *priscus*. Most *C*. *lavae* (70%) show simple overlap while a similar percentage of *C*. *magnipes* exhibit interdigitation.

When the frontals and parietals fail to meet along their medial borders, an opening in the skull roof results. This frontoparietal fontanelle is most pronounced in paedomorphic salamanders such as *Batrachoseps* [27] and *Thorius* [16]. None of the *Chiropterotriton* species examined here show frontoparietal fontanelles as large as members of those genera, but variation is evident (Table 2, Figs 2A–5A and 10). The fontanelle is relatively extensive in all *C*. *dimidiatus* as well as in a few (30%) *C*. *priscus*. The remainder of *C*. *priscus* show a reduced opening, as do all *C*. *lavae*. The most extensive development of the frontals and parietals is found in *C*. *magnipes*, in which obliteration of the fontanelle is found in 70% of the specimens examined.

Several bony processes were observed to project from the posterior region of many of the skulls examined (Table 2). The most unusual of these structures was seen in half of the *C*. *magnipes* specimens. In these animals, a flat, posterodorsally projecting, tab-like process was seen to emerge from each parietal at a level just anterior to the articulation with the otic capsule (Fig 2A). These processes were present in various states of development, from only slightly raised ridges to well-developed, quadrangular tabs. Three of the five animals possessing this structure did so asymmetrically, with only one process on either the right or left parietal (Table 5). Such a parietal process has been noted in plethodontids [4], but its functional significance is unknown.

Bony processes are present on the otic capsules of all species examined. In *C*. *dimidiatus*, *C*. *lavae*, and *C*. *priscus*, only one pair of processes was seen on any individual. These processes are cylindrical, arise from the dorsolateral surface of the capsule just anterior to the squamosal, and project laterally (Fig 3A). Such structures were present at an occurrence rate of 65% (number of observed occurrences/number of possible occurrences) in *C*. *priscus*, 30% in *C*. *lavae*, but only10% in *C*. *dimidiatus*. Their presence is mostly bilateral although some asymmetry was seen in *C*. *dimidiatus* and *C*. *priscus* (Table 5).

The same laterally projecting process was seen in all specimens of *C*. *magnipes*, in which it is often long and well developed. In addition, another pair of processes was seen in these animals. These were located more anteriorly on the otic capsule and, while also projecting laterally, were more tab-like in appearance. Therefore, all *C*. *magnipes* examined possessed two well-developed pairs of otic capsule processes (Fig 2A).

Squamosal processes were observed to occur at a rate of 35% in *C*. *magnipes* and unilaterally in one individual of *C*. *priscus*. These processes arise along the anterodorsal margin of the squamosal and project laterally in the same direction as, and nearly side by side with, the posterior most otic process (Fig 2A). Although squamosal processes are not unusual in other plethodontids [4], their presence in bolitoglossines has not been commonly noted. A posteriorly directed squamosal process or spur is found in all *Thorius* and *Oedipina* [4, 16, 26], but it is a different structure than the *Chiropterotriton* process described here.

One additional point concerning the squamosal is its orientation with respect to the rest of the skull. Normally, the jaw suspensorium (squamosal, quadrate, and palatoquadrate cartilage) extends ventrolaterally from the squamosal's dorsal articulation with the otic capsule. Therefore, the entire squamosal and at least a portion of the quadrate are visible in a dorsal view of the skull. In *Thorius*, Hanken [16] noted that the suspensorium descends ventrally from an articulation point on the underside of the otic capsule, rendering the entire structure no longer visible in dorsal view. The suspensoria of the species of *Chiropterotriton* examined here range from extreme horizontal to vertical orientations. *C*. *magnipes* has squamosals that extend far laterally and are fully visible in dorsal view (Fig 2). This almost horizontal orientation correlates with the broad and extraordinarily flattened skull shape of this species. The structure becomes decidedly more vertical as one progresses to *C*. *priscus*, to *C*. *lavae*, and finally to *C*. *dimidiatus*, where the angle is almost perfectly vertical and the structure almost disappears in dorsal view (Figs 3–5).

#### Ventral Skull

The most significant ventral structure seen in all four species of *Chiropterotriton* examined is a well-developed ventral "tab" formed by a ventromedial extension of the parietal (Figs 2B–5B). This structure appears to "wrap around" the braincase just posterior to the orbitosphenoid, an orientation that suggests a role in strengthening the skull. Such a structure is not unknown in plethodontids [4], but the extent of its development as observed here for *Chiropterotriton* is noteworthy.

Further evidence indicating the high degree of ossification of *Chiropterotriton* skulls is seen in the unfailing presence of well-developed opercular plates and columellae. Columellae in particular are often reduced or absent in neotropical genera. Wake [4] noted that members of *Chiropterotriton* have the best-developed columellae, and while some variation was seen in the species examined here, Wake's observation is confirmed.

Ventrolaterally, the orbitosphenoid contributes to a bony braincase, extending vertically from the parasphenoid and dorsally to the frontal and parietal. In *C*. *magnipes*, *C*. *priscus*, and *C*. *lavae*, the orbitosphenoid articulates solidly with the frontal and parietal. In *C*. *dimidiatus*, however, the orbitosphenoid falls short of these bones and a distinct gap is visible (Fig 5B). A similar situation exists in *Thorius*, where a membrane stretches across this gap [16].

The vomers in these *Chiropterotriton* are essentially as described by Wake [4]. The anterior tooth series extend along the posterior margins of the vomers and these teeth are well separated from the two, distinct posterior patches. Variation is seen in the lateral extent of the anterior teeth and the extent of the preorbital processes (Figs 2B–5B). All *C*. *magnipes* and *C*. *priscus* have well developed preorbital processes, with teeth extending almost to the lateral extent of these structures. Some (30%) *C*. *lavae* have shortened processes, and although most (70%) of the specimens have preorbital processes present, that portion of the bone is without teeth (Fig 4B). The entire preorbital process is absent in all *C*. *dimidiatus* (Fig 5B).

Counts of functional teeth (i.e. those teeth ankylosed to the bone) on the maxillae and premaxilla revealed several apparent trends (Table 3). The first was an increase in the average number of teeth with increasing species size. Thus *C*. *dimidiatus* had the fewest teeth, followed in order by *C*. *lavae*, *C*. *priscus*, and *C*. *magnipes*. This trend is strong for maxillary teeth, but decidedly less pronounced for premaxillary teeth. Low average premaxillary tooth numbers and overlapping ranges for *C*. *dimidiatus*, *C*. *lavae*, and *C*. *priscus* obscure any possible premaxillary patterns in these species. *C*. *magnipes*, however, had many more teeth than the other species, with more than twice the number of both premaxillary and maxillary teeth observed in the species with the next greatest number.

This trend toward increased numbers of teeth in larger species can be partially attributed to a simple correlated increase in the dental region of the maxillae and premaxilla, with a resulting increase in tooth loci and therefore functional teeth. This is not the entire explanation, however. The absolute size of individual teeth is also important, and in these species there is a trend toward smaller teeth in the larger species. This trend is especially evident in the extremes—*C*. *magnipes* has high numbers of extremely small [28] teeth (Fig 2B), whereas males of *C*. *dimidiatus* have very few, large, almost fang-like teeth (Fig 5B).

Examination of males and females separately revealed sexual dimorphism in both tooth number and tooth size. Females have more teeth than males in all species, while males tend to have individual teeth of larger size. Both of these dimorphic patterns are seen very strongly in *C*. *dimidiatus* and *C*. *lavae*, and to a lesser extent in *C*. *priscus* and *C*. *magnipes*.

Further sexual dimorphism is seen in *C*. *dimidiatus* and *C*. *lavae* in relation to the posterior extent to which teeth are found on the maxillae. The large teeth of males extend posteriorly only to about the posterior-most portion of the facial lobe of the maxilla, with occasional, very small teeth arising from the remainder of the bone (Figs 4B and 5B). Females possess smaller teeth in a regular pattern along almost the entire length of the maxilla. Such dimorphism is not seen in *C*. *priscus* and *C*. *magnipes*, in which the teeth extend almost to the posterior end of the maxillae in both sexes.

### Post-cranial Osteology

#### General Observations

Except for the structure of the feet, the post-cranial osteology was not studied in detail. In most instances, the remaining structures were similar across the four species studied. All unfailingly possessed 14 trunk vertebrae, the number typical of all neotropical genera except *Oedipina*. Ossification levels were similar in most structures. Only *C*. *dimidiatus* showed higher levels of ossification, evident in the articulating surfaces of the long bones, especially the proximal ends of the humerus and femur. Some mineralization was also evident in the hyobranchial skeleton of a single specimen of *C*. *dimidiatus*.

Tibial spurs are well developed in most plethodontid genera [4]. This structure was seen to be highly variable in the four species of *Chiropterotriton* studied here (Table 4). *C*. *magnipes* lacked tibial spurs altogether, although many (60%) did show a slight ridge-like structure in its place (Fig 11A and 11B). All *C*. *dimidiatus* showed a well developed, "detached" spur with a well-defined distal process rising away from the main body of the tibia (Fig 11C). Most (70%) *C*. *lavae* exhibit a similar spur morphology, with the remainder (30%) showing an "attached" spur, of which the distal process is joined to the tibia by a thin shelf of bone (Fig 11D). All *C*. *priscus* showed attached tibial spurs, but almost all (95%) of these were characterized by a foramen piercing the thin shelf of bone (Fig 11E).

#### Feet

**Fig 14:**
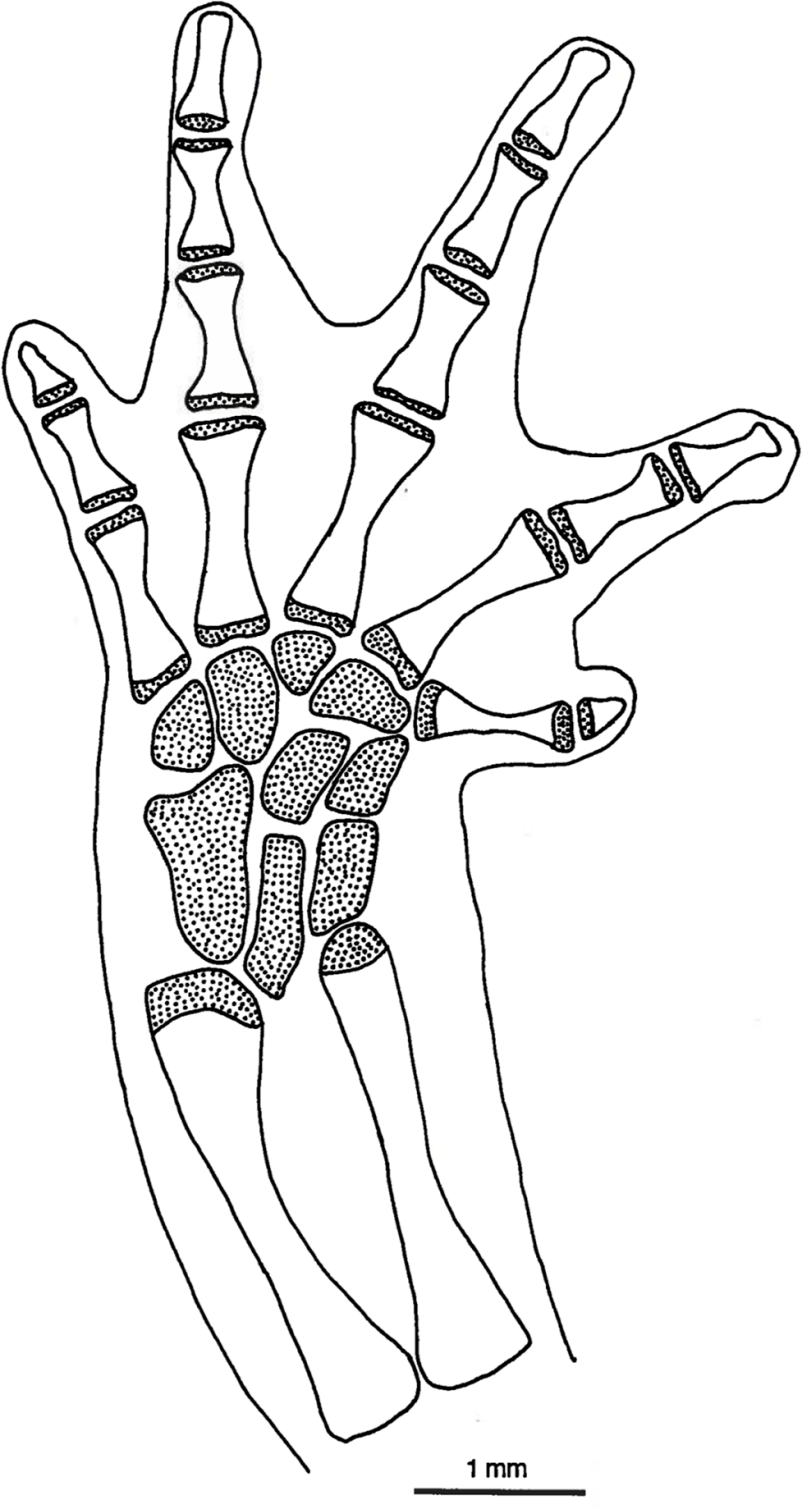
*Pseudoeurycea leprosa* foot. Drawing of left, rear foot of *Pseudoeurycea leprosa* (MVZ 147092), showing skeletal elements and the extent of interdigital webbing. Dorsal view; stippled areas represent cartilage. Elements as labeled in Fig 5.

The normal carpal and tarsal patterns for *Chiropterotriton* can be seen in Figs 6–9 and can be compared to the hypothesized but well documented ancestral plethodontid patterns [22, 29, 30]. The genus exhibits the ancestral plethodontid carpal morphology consisting of eight distinct cartilages: three proximal elements (ulnare, intermedium, and radiale), a centrally located centrale, a medial element “y”, and three distal elements (distal carpals 1–2, 3, and 4) (Figs 6A–9A) [4, 29]. The tarsus of *Chiropterotriton* also resembles the ancestral state in that there are typically nine distinct cartilages: three proximal elements (fibulare, intermedium, and tibiale), a centrale, element “y”, and four distal elements (distal tarsals 1–2, 3, 4, and 5) (Figs 6B–9B) [4, 29]. (Note: The single elements designated here as distal carpal 1–2 and distal tarsal 1–2 are equivalent to the basale commune; see [30] for terminology.) The *Chiropterotriton* tarsal arrangement differs from the ancestral plethodontid state, however, in the relative proportions of distal tarsals 4 and *5* and the articulation of distal tarsal *5* with the centrale. In the ancestral state, distal tarsal 4 is larger than distal tarsal *5* and essentially blocks its articulation with the centrale (Fig 14). In *Chiropterotriton*, the situation is reversed. Distal tarsal 5 is larger than 4 and articulates broadly with the centrale (Figs 6B–9B)

All but five of the forty individuals examined exhibited the typical *Chiropterotriton* carpus and tarsus. Three of these animals showed anomalous carpal arrangements, with the other two showing unusual tarsal morphology. In each case, the atypical morphology was unilateral in nature and was manifested as either a reduction or an increase in the number of carpal or tarsal cartilages. A reduced number of cartilages, a normal condition for some plethodontids [4, 12], is interpretable here as fusion of two elements into a single, larger element. For instance, in the carpus of one *C*. *priscus*, the ulnare and intermedium were fused to form a single cartilaginous element. Similarly, a specimen of *C*. *dimidiatus* exhibited a fusion of distal carpals 1–2 and 3. The addition of elements can cause the mesopodial arrangement to appear quite different from the normal pattern, making interpretation less straight forward (for discussion see [31]). Such unusual patterns were seen in one *C*. *priscus* tarsus, one *C*. *dimidiatus* tarsus, and one *C*. *dimidiatus* carpus. No anomalous carpal or tarsal arrangements were seen in either *C*. *magnipes* or *C*. *lavae* and no calcification of elements was observed in any of the four species.

Metacarpals and metatarsals showed little variation in *C*. *dimidiatus*, *C*. *lavae*, and *C*. *priscus*. In these species, the elements showed the typical hourglass shape and little specialized structure. In *C*. *magnipes* however, both metacarpals and metatarsals are widened to appear almost rectangular in shape (Fig 6). In addition, the first and fourth metacarpals and first and fifth metatarsals show unusual distal joint surfaces. The cartilaginous epiphysis not only covers the distal end of these elements, but also extends over the distolateral surface to form an expanded joint surface (Fig 6).

All four species showed the ancestral plethodontid pattern of four fingers and five toes and the primitive phalangeal formulae of 1-2-3-2 and 1-2-3-3-2 [4]. The terminal phalanges were expanded to some degree in all species, but this was most extreme in *C*. *magnipes*, where these elements appear to bifucate (Fig 6). The terminal phalanx of the first digit, usually reduced and unspecialized in morphology in plethodontids, showed unusual form in *C*. *magnipes*. In this species, this phalanx was well developed in both the hands and feet, showed a tendency toward bifurcation, and was consistently seen to have a small cartilaginous tip (Fig 6).

## Discussion

### Osteological Characters and Monophyly

E.H. Taylor, in his original 1944 description of *Chiropterotriton*, diagnosed the genus as he then understood it [32]. He presented two lengthy paragraphs of phenotypic characters present in the genus. Unfortunately, many of these characters were not present in all of the species known at the time, and those that were characteristic of all species were more often than not found in members of other genera as well. This reflects systematic practice of that time. *Chiropterotriton* was indeed a recognizable unit, but the importance of using synapomorphic characters to define taxa and clades was not recognized, and detailed knowledge of plethodontid morphology and biology was limited. Use of genetic data was well in the future.

In their 1983 review of neotropical plethodontids [1], Wake and Elias selected a subset of characters that could be treated discretely, with evolutionary direction deduced on the basis of outgroup analysis. The phylogenetic analysis performed using these characters allowed for determination of the monophyletic status of the neotropical genera as well as the generation of phylogenetic hypotheses of relationship among the genera. This analysis indicated that *Chiropterotriton*, as originally defined by Taylor, was not monophyletic and was in fact made up of at least three clades. Several species were therefore removed from *Chiropterotriton* and placed into two new genera—*Dendrotriton* and *Nototriton* (the one *Chiropterotriton* species they assigned to this genus as *Nototriton nasalis* is now placed in another recently designated taxon, *Cryptotriton* [33]).

The phylogenetic standing of the revised genus of *Chiropterotriton* has since been studied by a number of workers using various methods of morphological and genetic analysis [34–37]. One thing that all of these studies confirm is the monophyletic status of the genus (as restricted by Wake and Elias [1]), this in spite of the diversity of body forms and paucity of synapomorphic morphological characters.

The single morphological synapomorphy defining *Chiropterotriton* is the derived tarsal arrangement characteristic of this genus. The present osteological analysis reveals no clear additional synapomorphies, although two characters are worth noting. The first is the presence of large, well-developed septomaxillary bones in all species of the genus. What is unique here is the size of these elements, not merely their presence. Septomaxillaries occur in many other genera, and their presence is an ancestral condition and therefore a symplesiomorphy. In no other neotropical genus, however, do *all* member species exhibit these bones, and at such a consistently large size. The second osteological character of note is the presence of well-developed parietal tabs visible ventrally in the skull (Figs 2B–5B). Such tabs are seen in other genera (e.g. *Bolitoglossa* and *Oedipina*), but they are especially well developed in the *Chiropterotriton* species examined here. Systematic analysis of the remaining species of *Chiropterotriton* and comparison with other neotropical genera is necessary to test whether the level of development of these characters might be useful in further defining the genus. If so, these characters, when combined with the tarsal synapomorphy, would add further morphological evidence to the monophyly of *Chiropterotriton*, already so well supported by molecular evidence.

### Intraspecific Variation

Variation of osteological characters within amphibian species has been investigated in relation to adaptation and constraint [16, 38]. Hanken [16] stated that in several species of *Thorius*, levels of osteological variation seen in several characters had to be considered extreme. Specifically, he cited three characters—premaxilla development, nasal-maxilla overlap, and prefrontal development (measured in terms of prefrontal-maxilla articulation)—in which "the range of intrapopulational variation in a given species of *Thorius* may nearly match the range of variation of each character in other plethodontid genera".

While the species of *Chiropterotriton* examined here appear to match the species of *Thorius* in the levels of intraspecific variation seen in nasal-maxilla articulation and prefrontal-maxilla articulation, it is notable that they show less variation in premaxilla development (no *Chiropterotriton* showed fusion of the ascending processes). That no fusion of these processes is seen in *C*. *dimidiatus* (Fig 5) is in contrast to Wake [4], who noted such fusion in *C*. *dimidiatus* and attributed the condition to paedomorphic influences. Earlier in the same work (p. 12) Wake stated that no such fusion had been observed in the genus as currently diagnosed and studied. This seeming contradiction was apparently due to a single specimen with fused frontal processes found during the later stages of Wake's study [4] (unpublished data). Thus, while such fusion does occur, it is apparently in low frequency.

Intraspecific variation levels for *Thorius* and the *Chiropterotrito*n species examined here are similar for the presence or absence of septomaxillary bones, although the trends are opposite in the two genera. Septomaxillae are generally present in *Chiropterotriton* species, while in *Thorius*, most members of the genus lack these elements. The rate of septomaxilla occurrence in *C*. *dimidiatus* is lowest (35%) of the species examined here. It is noteworthy that no septomaxillae were found in the five specimens of *C*. *dimidiatus* examined earlier by Wake [4]. This absence was attributed to paedomorphosis [4], and studies of other neotropical plethodontids that lack septomaxillae have shown that paedomorphosis is indeed a probable explanation for reduction and loss of these late developing bones [11, 16]. In this light, it is notable that these elements are most often absent in the miniaturized *C*. *dimidiatus* and when they do occur, they are often reduced in size and are asymmetric (Table 5).

Hanken [16] calculated frequencies of right-left asymmetry, with levels as high as 35%. *Chiropterotriton* showed an asymmetry level of 60% for nasal-maxilla articulation in *C*. *dimidiatus* (Table 5). *Chiropterotriton* asymmetry levels also are somewhat higher than *Thorius* in nasal-prefrontal articulation and of a similar magnitude in the presence of septomaxillae.

Hanken [16] hypothesized that the high levels of intraspecific variation he observed in *Thorius* represented "a 'by-product' of skeletal reduction", and that it reflected "a relaxation of functional constraints that serve to stabilize the morphology of individual elements of larger salamanders". Except for *C*. *dimidiatus*, the species of *Chiropterotriton* examined here are "larger salamanders”, yet similar levels of intraspecific variation are observed.

Perhaps these highly variable characters are simply not subject to rigid functional constraints in plethodontids, no matter what their size. It should be noted, however, that while levels of variation are generally similar in species of both genera, within *Chiropterotriton*, the smallest species, *C*. *dimidiatus*, is slightly more variable than the other species examined, lending some support to Hanken's miniaturization hypothesis. Further testing must await the examination of these same characters in other groups containing species of various sizes.

### Interspecific Variation

#### Phylogeny

Most of the interspecific variation seen in the osteological characters studied here is proportional. For instance, within each of the four species, some individuals possessed septomaxillary bones while others did not. The observed variation is therefore not the result of these elements existing in some species and not others, but rather to what extent they exist in each species. In this particular case, *C*. *dimidiatus* is proportionally quite different from the other three species in that septomaxillaries are more often absent. Such proportional variation is not unusual in plethodontid salamanders [11, 16], and although not as useful as discrete variation for generating phylogenetic hypotheses, it can be of value in understanding morphological transitions between species, especially when interpreted in conjunction with phylogenetic hypotheses generated by other data sets.

While most osteological variation seen here in *Chiropterotriton* is proportional, several qualitative differences were found, and these can be divided into two types of differences: fixed and polymorphic. Fixed qualitative differences are those in which one character state was found in all individuals of one species or group of species while the remaining species exhibited one or more different character states. Polymorphic qualitative differences are those in which a character state, while unique to one species or group of species, is not seen in all of the individual members. Fixed differences can surely be used in a phylogenetic analysis. Polymorphic differences are more difficult to deal with, for it is unclear how frequently a character state must be observed in a species before it is scored as being present. Wake and Elias [1] dealt with this issue at the intergeneric level and opted for a conservative approach by eliminating such polymorphic characters from their analysis.

Four fixed qualitative differences were observed among the osteological characters examined here. For instance, all individuals of *C*. *magnipes* had two processes arising from the otic capsules. This condition was not seen in any individual of any of the other species. Similarly, all *C*. *magnipes* were found to lack tibial spurs while *C*. *dimidiatus*, *C*. *lavae*, and *C*. *priscus* individuals possessed a spur in some form. Two fixed qualitative differences separate *C*. *dimidiatus* from the other species. All individuals of *C*. *dimidiatus* were characterized by the absence of the preorbital processes of the vomer and by the distinct separation of the orbitosphenoid from the frontal and parietal (these conditions are seen in other bolitoglossines and are another example of the extensive homoplasy within the tribe). All individuals of the other species possessed preorbital processes and showed solid articulations among the bones making up the braincase.

Although qualitative and fixed, these four characters appear to provide little or no phylogenetic information. In each case, the derived condition characterizes only one species. These autapomorphies are useful in affirming and defining the monophyletic status of *C*. *magnipes* and *C*. *dimidiatus* but say nothing about the interspecific relationships among the four species. It is possible, however, that upon examination of more *Chiropterotriton* species, these characters could prove to link different taxa.

Though of questionable phylogenetic value, several polymorphic qualitative differences warrant mention, for they too confirm the unique status of two of the species examined. Five such polymorphic qualitative traits define *C*. *magnipes* as a unique entity. For instance, although present only 35% of the time in *C*. *magnipes*, parietal processes (character 9) were found only in that species. The remaining four characters separating *C*. *magnipes* from the other species include the high degree of overlap seen between the nasal and premaxilla (character 2), the interdigitation of the right-left frontal (character 6) and parietal (character 7) pairs, and the complete absence of a frontoparietal fontanelle (character 8). The percentage in which these unique character states were present in *C*. *magnipes* ranged from 40 to 70%. The only other species to show such a unique character state was *C*. *priscus*. The tibial spur of *C*. *priscus* differed from all other species in being both attached to the tibia with a thin shelf of bone and having that shelf pierced by a distinct foramen. Only one *C*. *priscus* showed a unilateral variation from such a morphology.

#### Skull Robustness

Of the 18 osteological characters examined, 15 (1–15) are related to skull robustness as measured by the extent of bone development—more bone resulting in a more robust skull. This robustness is reflected in differing levels of skull bone articulation (characters 2–7, 13), the extent of bony processes (characters 9–12), the size of the frontoparietal fontanelle (character 8), the number of functional teeth (characters 14–15), and the simple presence or absence of septomaxillary bones (character 1). When these characters are individually scored from least to most robust and the scores totaled, the overall character of skull robustness results (Table 6). *C*. *dimidiatus* clearly has the least robust skull in almost all aspects, while *C*. *magnipes* consistently shows the most extensive bone development and most robust skull of the group. *C*. *lavae* and *C*. *priscus* show similar levels of skull robustness intermediate to *C*. *magnipes* and *C*. *dimidiatus*. A superficial pattern of skull robustness emerges—low robustness, intermediate robustness, and high robustness.

**Table 6:** Skull robustness.

Character	Species			
	*C*. *magnipes*	*C*. *priscus*	*C*. *lavae*	*C*. *dimidiatus*
1. Septomaxilla development	2	4	2	1
2. Nasal-premaxilla articulation	4	1	3	2
3. Nasal-maxilla articulation	4	3	2	1
4. Nasal-prefrontal articulation	4	3	1	2
5. Nasal-frontal articulation	4	4	4	1
6. Frontal-frontal articulation	4	1	3	2
7. Parietal-parietal articulation	4	2	3	1
8. Frontoparietal fontanelle	4	1	3	1
9. Parietal process development	4	1	1	1
10. Otic process development	4	3	2	1
11. Squamosal process	4	3	1	1
12. Vomer preorbital process development	4	4	2	1
13. Orbitosphenoid-frontal-parietal articulation	4	4	4	1
14. Premaxilla functional tooth number	4	2	3	1
15. Maxilla functional tooth number	4	3	2	1
**Skull robustness score**	**58**	**39**	**36**	**18**

1 = less robust; 4 = more robust

### Osteological Patterns and Evolutionary Processes

#### Potential Explanations of Pattern

How can the skull robustness pattern seen here be explained, and what can we learn about morphological evolution in *Chiropterotriton*? Four potential explanations are presented here as separate hypotheses that might result in the observed pattern. These hypotheses are not exclusive of each other, nor are they presented as alternative hypotheses to imply that only one is the correct explanation. Two, three, or potentially all four might contribute to the skull osteological variation. Treatment as separate hypotheses is meant only to separate out potential factors and to potentially give some indication of relative importance.

#### Hypothesis 1

The variation in skull osteology seen in these four species of *Chiropterotriton* can best be explained by their phylogenetic relationship.

In a sense, this is undoubtedly true, for knowledge of phylogeny entails knowledge of history, and if the history of the genus were known, then we would at least be in a better position to know how *Chiropterotriton* morphologies evolved.

#### Prediction 1

**Fig 15:**
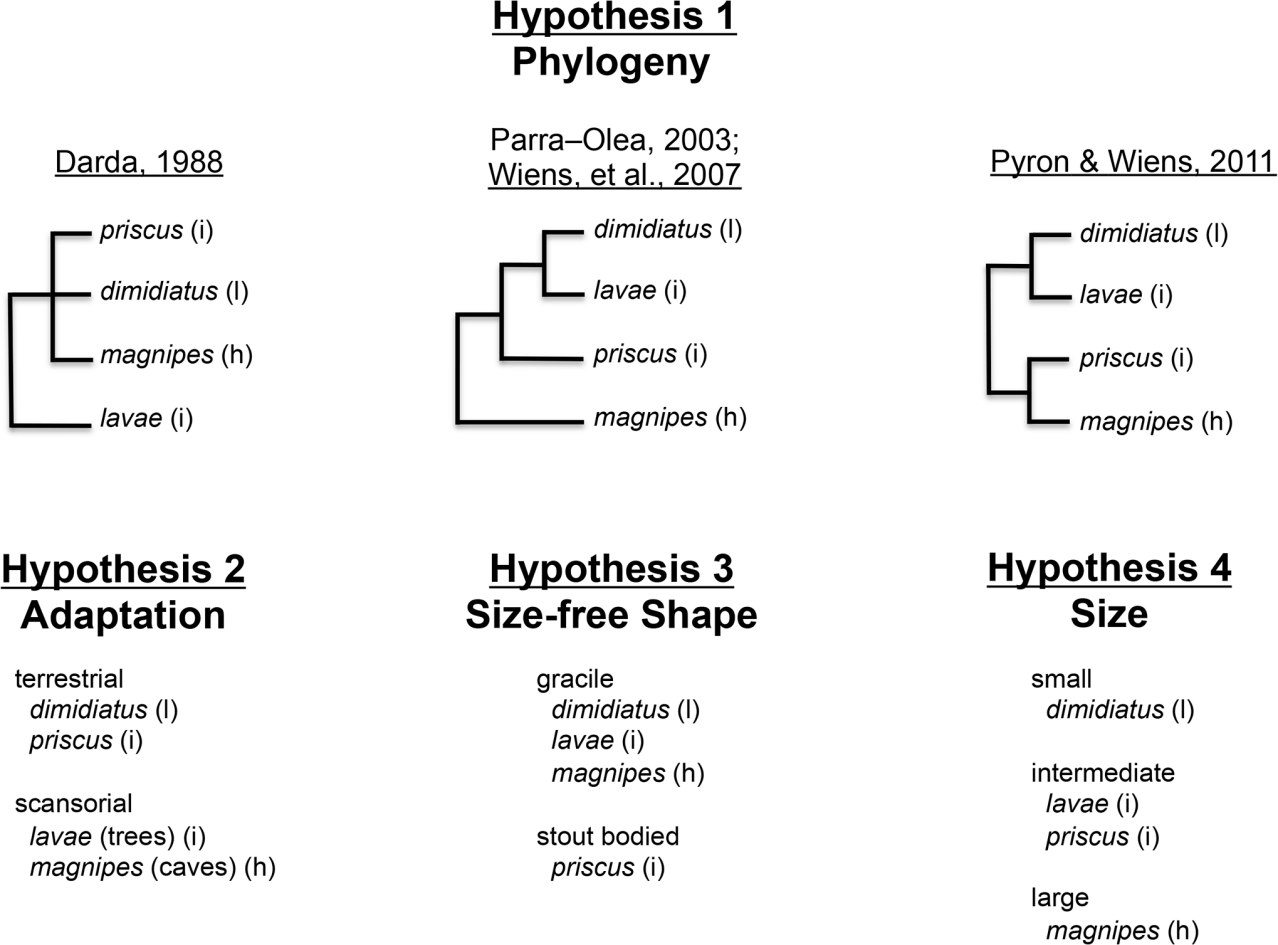
Skull robustness hypotheses. Four hypotheses representing potential explanations of the pattern of skull robustness seen in the four species of *Chiropterotriton* examined in this study and the morphological groupings predicted by each hypothesis. Skull robustness: l = low, i = intermediate, h = high.

Unfortunately, we do not know the history of *Chiropterotriton*, and attempts at resolving the interspecific phylogenetic relationships have not proved robust [33–36]. Three of four proposed phylogenies would suggest that *C*. *dimidiatus* and *C*. *lavae* should be the most alike osteologically, with *C*. *priscus* and *C*. *magnipes* either clustering together or having separate sister relationships (Fig 15).

#### Hypothesis 2

The variation in skull osteology seen in these four species of *Chiropterotriton* can best be explained by adaptation to specific habitats.

*C*. *priscus* and *C*. *dimidiatus* are strictly terrestrial animals whereas *C*. *magnipes* and *C*. *lavae* are scansorial. *C*. *lavae* is found in bromeliads in trees, and *C*. *magnipes* is a cave dweller, and has been found on surfaces well up from the cave floor. The assumption implicit in this hypothesis is that different biomechanical demands would be predicted for animals occurring in these two habitats, resulting in morphological differences.

#### Prediction 2

Two species pairs should be recognizable osteologically. The terrestrial *C*. *priscus* and *C*. *dimidiatus* and the scansorial *C*. *magnipes* and *C*. *lavae* (Fig 15).

#### Hypothesis 3

The variation in skull osteology seen in these four species of *Chiropterotriton* can best be explained by size-free shape differences.

Darda [39] showed that *C*. *priscus* was the most different of the four species in terms of size-free shape. If size-free shape is an important factor in the overall morphological differences seen in these animals, then those species similar in shape should show similar osteological patterns.

#### Prediction 3

C. *magnipes*, *C*. *lavae*, and *C*. *dimidiatus* should be the most alike osteologically, with *C*. *priscus* being the outlier (Fig 15).

#### Hypothesis 4

The variation in skull osteology seen in these four species of *Chiropterotriton* can best be explained by overall size differences.

Variation in size among these species is great. Adults of each species can be distinguished on the basis of size alone. If size is also important in producing osteological variation, such variation should follow the same pattern as adult size.

#### Prediction 4

Osteological variation should reflect the progression in size seen in these species, from the extremely small *C*. *dimidiatus* on one end of the size spectrum to the very large *C*. *magnipes* on the other. *C*. *lavae* and *C*. *priscus* should cluster between these extremes. (Fig 15).

Of these four hypotheses, only Hypothesis 4 predicts the exact pattern seen in the skull robustness of the four species, suggesting that the size differences seen among these four species of *Chiropterotriton* are an important factor in generating the observed osteological variation. If size change is an important force at this level of variation, it also may be an important factor in the evolution of the overall diverse morphologies seen in *Chiropterotriton*. Therefore, the morphological transitions between these extreme members of *Chiropterotriton* might be explained to a large degree by size change over evolutionary time.

#### Size and Heterochrony

Evolutionary size change is a repeated phenomenon in plethodontids and salamanders in general and is associated with heterochrony [6, 40]. Paedomorphic changes appear to be the predominant mechanism for size reduction in the genus *Thorius* [12, 13, 15, 16], and it seems likely that size reduction in *Chiropterotriton* (e.g. *C*. *dimidiatus*) has also entailed such heterochronic change. Peramorphosis has been cited as potentially important in the evolution of certain characters in the arboreal plethodontid, *Aneides lugubris*, the largest member of the genus *Aneides* [25, 41]. Such a process may also be important in the evolution of certain osteological features seen in *C*. *magnipes*, such as the interdigitating articulations between frontal and parietal pairs, the absence of a frontoparietal fontanelle, the presence of parietal processes, and the presence of more than one process on each otic capsule.

Heterochrony is often a "global" phenomenon in salamanders. Thus, one expects a suite of morphological features, not just a single character, to be affected [10, 42]. The suite of osteological characters associated with skull robustness examined here seems to track together as expected with heterochronic change and strongly suggests that morphological evolution in *Chiropterotriton* is to a large degree due to developmental perturbations similar to those seen in other plethodontid lineages.

#### *Chiropterotriton*—a microcosm of plethodontid homoplasy

Homoplasy has been a major theme in plethodontid evolution, as well as salamander evolution in general [6, 18, 43]. Wake and Larson [20] concluded that in plethodontids, the "system of developmental transitions…produces a finite set of possible evolutionary transitions, the components of which are observed repeatedly during phylogenesis". These components are manifested in the form of parallel morphologies that are repeated within and among clades. Such homoplasy is common in plethodontids, and while it has been a confounding factor in understanding the *pattern* of plethodontid evolution [1, 6, 17], it may prove to be important in understanding the *process* of evolutionary change [44].

If well understood examples of homoplasy are indeed the crucibles within which our understanding of morphological evolution can be enriched, *Chiropterotriton* offers abundant opportunity. Within this well-defined genus are examples of morphologies seen in other plethodontid clades. In a sense, *Chiropterotriton* appears to be a microcosm of much of what we see in the entire family Plethodontidae.

#### Webbed Feet

Doubtless, the most characteristic feature of *Chiropterotriton magnipes* is that from which it derives its name—the large, fully webbed feet. While these structures are indeed impressive, they are far from unique. Numerous members of the genus *Bolitoglossa* show similar modifications in foot morphology [2, 45–47]. This aspect of *Bolitoglossa* morphology has been studied in detail [10, 21, 48].

Alberch [10] not only examined the osteology of such feet, but also conducted experiments aimed at understanding the functional, biomechanical, and adaptive features of such highly modified structures. He proposed two morphological modes in *Bolitoglossa*—Mode 1 and Mode 2. In Mode 1, he hypothesized that selection apparently acted to produce webbed feet, which are used as suction cup devices, by favoring feet with relatively large surface area and expanded terminal phalanges. In Mode 2, animals exhibit small body size and fully webbed feet, but show reduction in phalanx size and number, a morphology apparently resulting from selection for small overall size with attendant paedomorphosis. In both modes, the resulting feet are fully webbed, but Mode I animals tend to be relatively large (e.g. *Bolitoglossa mexicana* group) with large feet, while Mode 2 animals are smaller with small feet (e.g. *B*. *rufescens*).

More recently, Jaekel and Wake [21] have found evidence that it is more likely that *all Bolitoglossa* arose from common ancestor with webbed feet produced by paedomorphosis, and that reduced webbing is a derived state. Moreover, growth trajectories of webbed and unwebbed species of *Bolitoglossa* are identical, and mathematical modeling shows that interdigital webbing in *Bolitoglossa* does not increase to a degree that improves attachment to the substrate. While this evidence suggests that adaptation has not shaped *Bolitoglossa* webbing as suggested by Alberch’s Mode 1, Jaekel and Wake found *C*. *magnipes* to be a different story.

Like some of the fully webbed *Bolitoglossa* that are scansorial and can be found in bromeliads high in trees or foraging at night on large smooth leaves in the tropical forests [10], *C*. *magnipes* is also a scansorial animal, although instead of inhabiting trees or vegetation, this salamander appears to be confined to caves and sinkholes at lower elevations of the Sierra Madre Oriental in southern San Luis Potosi and eastern Queretero. *C*. *magnipes* has been collected high on vertical cave walls and even upside-down on cave ceilings and overhangs.

The large body size, fully webbed feet, and scansorial lifestyle suggest that *C*. *magnipes* might fall into Alberch's adaptive Mode 1, and the osteological evidence seen here is consistent with this. *C*. *magnipes* exhibits distally expanded terminal phalanges in both hands and feet (Fig 6). Alberch's experimental results suggested that these structures allow large, webbed species to produce a more effective seal along the rim of the webbing, thereby producing a more efficient suction and increasing the animal's ability to cling to a surface. In *C*. *magnipes* even the terminal phalanges of the first digits appear somewhat expanded or bifurcated distally. The distal tip of this element is often cartilaginous.

Other osteological modifications of the *C*. *magnipes* foot, possibly associated with increased suction efficiency, are seen in the metacarpals and metatarsals. These elements appear dorsoventrally flattened and almost rectangular in shape (Fig 6), as opposed to an hourglass shape seen in other species. Such flattened and broad elements may allow the foot to be placed perfectly flat on a smooth surface. By placing more area of the ventral foot surface in close contact to the substrate, any adhesive forces of attachment would be maximized. Similarly, air space between the foot surface and substrate would be minimized resulting in a higher negative pressure if a suction is generated by muscular contraction as proposed by Alberch [10].

The morphology of metacarpo-phalangeal joints 1and 4 and metatarsophalangeal joints 1 and 5 is also potentially important. This feature is especially clear in Fig 6, in which the metacarpal articular cartilages of these joints can be seen to extend not only along the distal end of these elements, but also along the distolateral surface of the bones. These joints would appear to allow lateral abduction of the phalanges distal to them. Such abduction would effectively stretch the webbing taut and might be instrumental in increasing the foot surface area in contact with a cave wall and perhaps in forming an effective seal against the cave substrate.

It appears that the large, fully webbed feet of *C*. *magnipes* fit well with Alberch's Mode l, in which the evolution of such structures is seen as the result of selection for increased suction efficiency. Alberch predicted that the evolution of a Mode 1foot morphology would result in "changes from the ancestral allometric patterns of growth in foot surface in relation to body weight". Such allometric growth in *C*. *magnipes* has now been shown by Jaekel and Wake, resulting in a “species with very large feet compared with its body size, suggesting adaptation to its lifestyle.”

So, yes, we have a case of homoplasy—large, webbed feet in two distinct lineages (*Bolitoglossa* and *Chiropterotriton*). More importantly, in addition to this *pattern* of evolution, we understand something of the evolutionary *processes* involved. In *Bolitoglossa*, it appears that we have a global developmental change that results in a common webbed foot morphology with little adaptive significance, while in *Chiropterotriton*, selection has acted to modify the foot morphology of this single species with specific adaptive consequences.

#### Miniaturization

*Chiropterotriton dimidiatus* represents the small size extreme for the genus. Miniaturization has been studied for members of the genus *Thorius*, another neotropical plethodontid genus with members that are among the smallest, extant, tailed tetrapods [12, 13, 15, 16]. Using *Thorius* as a model, Hanken [16] proposed a null hypothesis characterizing the morphology of miniaturized taxa, and suggested its use in comparative analysis of other apparently miniaturized animals as well as evaluation of possible mechanisms producing such morphological change.

The first of the three characterizations proposed in this null hypothesis of miniaturization is the "precocious truncation of development relative to the presumed ancestral ontogeny which produces a reduced, paedomorphic morphology". In *Thorius* such morphology is evident in the extensive frontoparietal fontanelles, extreme reduction in the bony elements of the nasal region (specifically the nasals, prefrontals, and septomaxillae), and reduction or absence of maxillary teeth. While not as extreme as in *Thorius*, *C*. *dimidiatus* also shows enlarged frontoparietal fontanelles, a reduction in the occurrence of septomaxillary bones, and a reduction in maxillary tooth number. Other probable paedomorphic characters of *C*. *dimidiatus* are the absence of preorbital processes on the vomers and the lack of solid articulation between the orbitosphenoid and the frontal and parietal bones. The extreme reduction of nasal and prefrontal bones seen in *Thorius* is not evident in *C*. *dimidiatus*. In fact, these elements are well developed and ossified.

The second feature of Hanken's null hypothesis is that an increased level of variability should be observed in miniaturized forms. As already discussed, the intraspecific variation in osteological features for *Chiropterotriton* is in many cases at a level at least as high as *Thorius* and in some instances even greater. This high level is not confined, however, to *C*. *dimidiatus* although levels may be slightly higher than in the other species examined, especially in right-left asymmetry (Table 5).

The third and final feature characterizing miniaturization, according to Hanken, is the presence of unique morphological novelties. These novelties may be functional in nature of merely "secondary consequences of physical rearrangements". In *Thorius*, novelties in cranial osteology appear to be mainly such secondary consequences and are due to cranial distortion effected by a relatively large brain and sense organs [14]. Three novelties in the cranial morphology of *Thorius* were noted by Hanken [16]: 1) the vertical orientation of the jaw suspensorium, 2) presence of a posteriorly directed squamosal process or spur, and 3) an anteriorly constricted braincase. Only the first of these is seen in *C*. *dimidiatus* and such an extreme vertical orientation is not seen in the other *Chiropterotriton* species examined. Other morphological novelties were not observed in *C*. *dimidiatus*.

Although this comparison of *C*. *dimidiatus* to *Thorius* and the features predicted of such miniaturized animals suggests differences between what might be expected morphologically and what is actually observed, the homoplastic *pattern* of extreme small size seems clear. But what of *process*?

In a study of *Thorius* appendicular skeletal morphology, Hanken [13] identified the probable mechanism by which members of this genus attain their miniaturized form. Carpal and tarsal elements are typically cartilaginous throughout the entire life of a plethodontid salamander. In adult *Thorius*, however, these elements are ossified. Apparently, the ossification of these elements as well as an increase in the degree of long bone ossification cause a much-reduced rate of growth after sexual maturity with the resulting miniaturized morphology characteristic of the genus. This cannot explain the reduced size of *C*. *dimidiatus* (which is, however, larger than most *Thorius)* in which mesopodial elements remain cartilaginous. So while *C*. *dimidiatus* does show the parallel morphological complex of miniaturization with members of *Thorius*, different mechanisms are responsible and remain unknown for *C*. *dimidiatus*.

#### Body Shape

**Fig 16:**
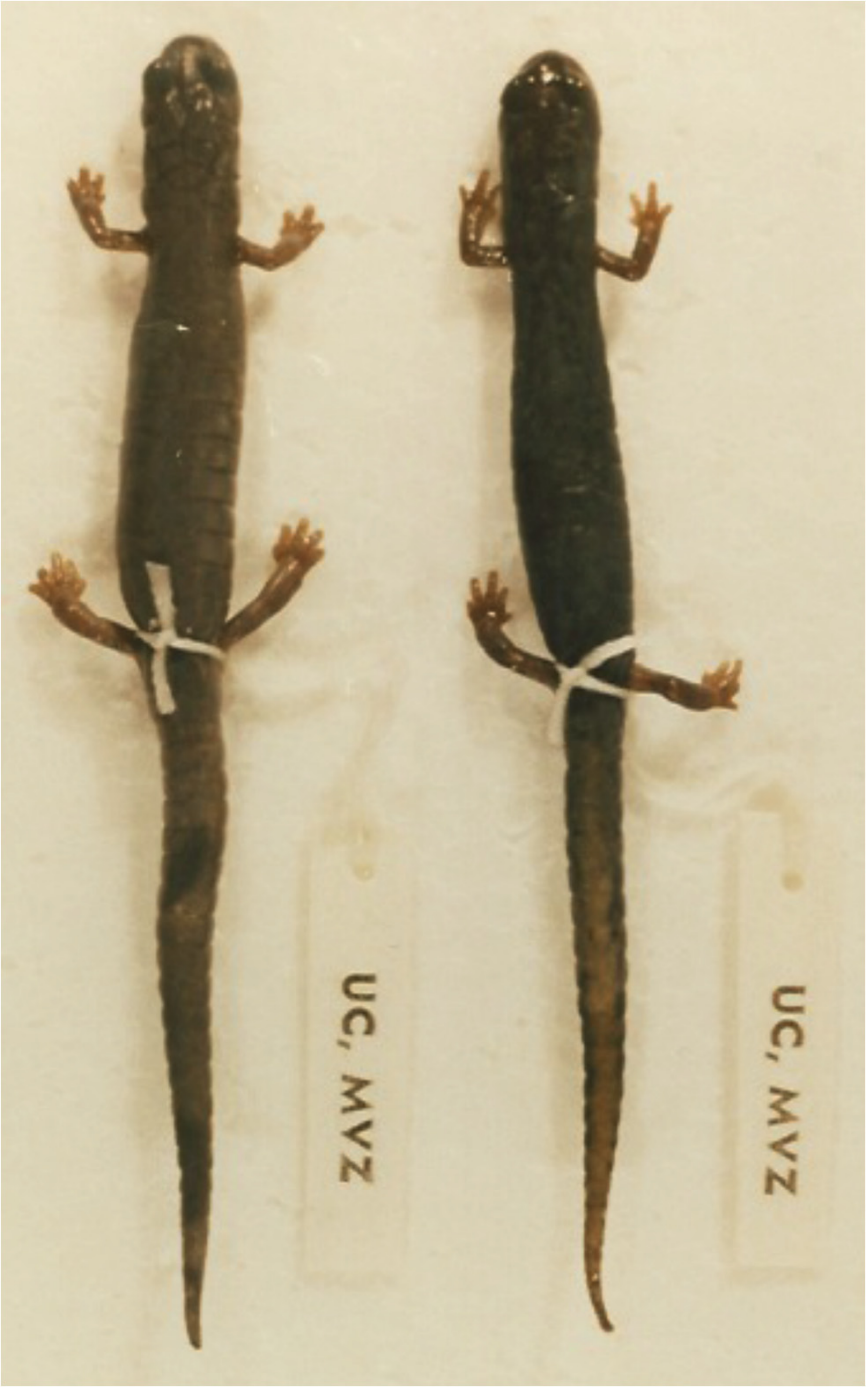
*Chiropterotrition priscus* body shape. Photograph showing the strikingly similar external appearance of (a) *Chiropterotrition priscus* (MVZ 138885) and certain members of the genus *Pseudoeurycea* [(b) *P*. *leprosa* (MVZ 132796)].

*Chiropterotriton priscus* is an atypical member of *Chiropterotriton* in terms of shape, in that it resembles members of other genera in general form [39]. This species has a stout body, relatively short limbs, a thick tail, and generalized external foot morphology. While these characters distinguish *C*. *priscus* from the rest of *Chiropterotriton*, they combine to produce an external form strikingly similar to many members of the genus *Pseudoeurycea* (Fig 16). The resemblance between *C*. *priscus* and *Pseudoeurycea* is so strong that when the species was originally described by Rabb [23], he made detailed comparisons not only to other members of *Chiropterotriton*, but also to *Pseudoeurycea galeanae*, which occurs in the same region of Nuevo Leon near Cerro Potosi. Rabb concluded that the new species was "a primitive and generalized species of *Chiropterotriton*" and not a member of the *cephalica* group of *Pseudoeurycea* to which *P*. *galeanae* belonged.

Subsequent workers have noted this parallelism in external morphology between *C*. *priscus* and *Pseudoeurycea*. Wake and Lynch [2] cited this parallelism as partial evidence in suggesting that *C*. *priscus*, along with *P*. *galeanae* and *P*. *cephalica*, might be the extant representatives of "the ancestors of the entire tropical assemblage of bolitoglossines", a view since complicated by the discovery of *Nyctanolis pernix* [22] and subsequent molecular data. Wake and Lynch hypothesized that the similarities between *C*. *priscus* and some *Pseudoeurycea* are actually retained generalized and ancestral anatomical features.

Confirmation of Rabb's original placement of *C*. *priscus* comes from much molecular work, which shows *C*. *priscus* nested well within the genus [34–37, 49]. Additionally, *C*. *priscus* possesses the *Chiropterotriton* tarsal synapomorphy unique among neotropical plethodontids, thus cementing this species' position as part of this monophyletic genus and reemphasizing its parallel external appearance to certain *Pseudoeurycea*.

#### Tarsal arrangement

*Chiropterotriton* is unique among tropical salamanders in having a reorganization of the tarsal elements. Distal tarsal 5 is larger than distal tarsal 4 and articulates with the central [4, 1]. This arrangement is found elsewhere only in *Aneides*, an unrelated plethodontid, and therefore another example of homoplasy [29].

Most species of *Chiropterotriton* are arboreal or scansorial, as are those of *Aneides*, which led Wake to propose that the evolution of the rearrangement constitutes a macroevolutionary transition [50]. The new arrangement is favorable biomechanically in that it directs all lines of force in straight lines from the digits, evenly through the distal tarsals, to the centrale. This enables species in these genera to greatly expand the span of digital extension, enabling grasping, and facilitating scansorial activity. This would be particularly important in *C*. *magnipes*, which is the only species of the numerous tropical salamanders known to have adapted to a cave existence.

**Fig 17:**
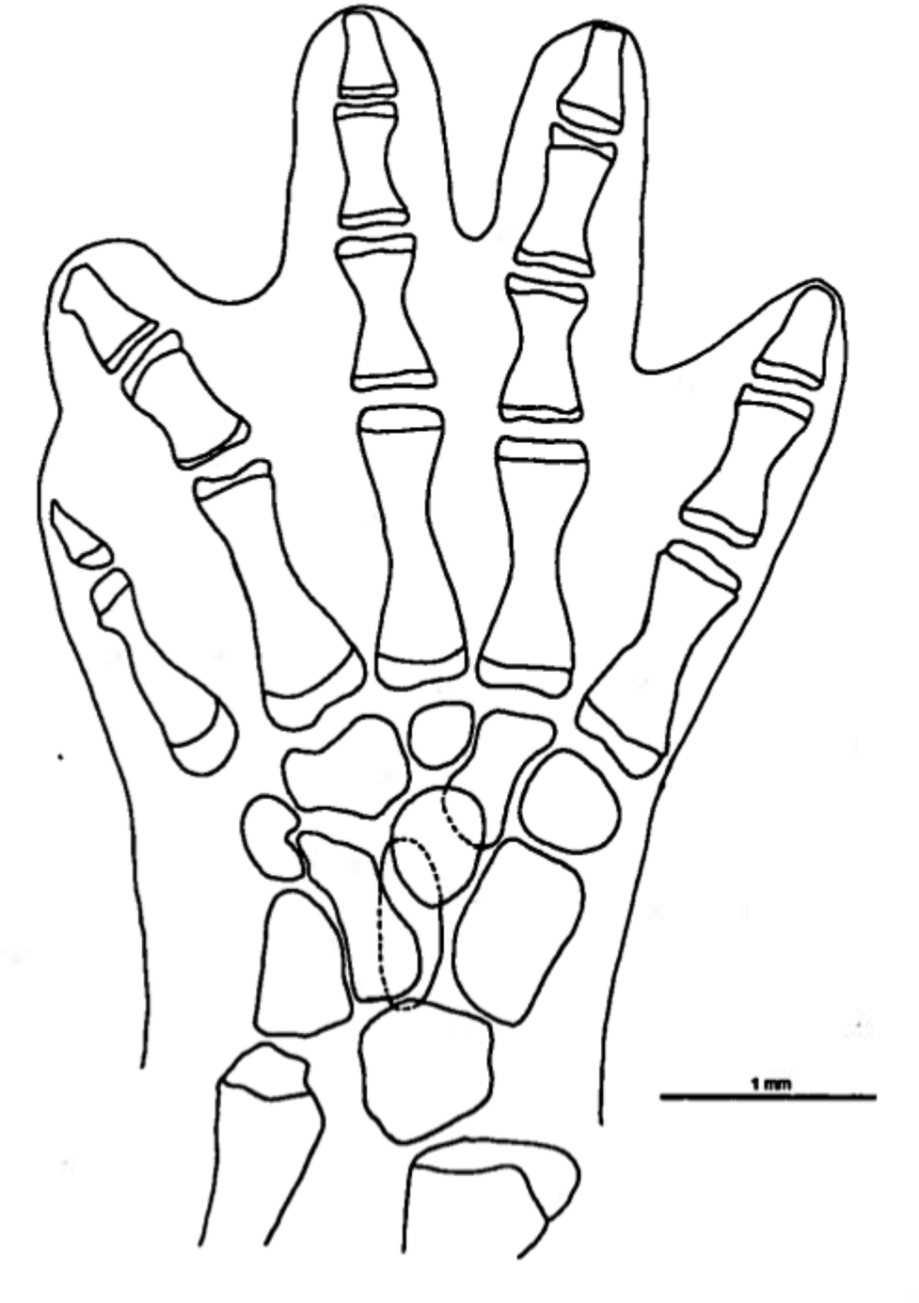
Anomalous *Chiropterotriton priscus* foot. Anomalous morphology of the right, hind foot of a single specimen of *Chiropterotriton priscus* (MVZ 138883). Dorsal view; distal tarsals labeled.

In this light, we note one specimen of *C*. *priscus* examined in this study that exhibited an anomalous situation in the tarsus of the right hind leg (Fig 17). Instead of the typical nine tarsal elements usually seen in the tarsus, this single foot contained eleven cartilaginous elements. While the precise homologies for the proximal and central rows are unclear, distal tarsals 3, 4, and 5 appear to be homologous to these same elements in a typical *Chiropterotriton* foot. The most striking feature is the large size and elongate shape of distal element 4, a size and shape similar to that found in the ancestral pattern found in other plethodontids, including *Pseudoeurycea* (Fig 14).

Although this aberrant *C*. *priscus* foot is not a perfect reproduction of the "normal" ancestral pattern, the appearance of an enlarged distal tarsal 4 is enough to suggest a reversal to the ancestral plethodontid morphology. The occurrence of such atavisms has been noted in other salamanders and suggests the retention of ancestral genetic developmental systems that are capable of producing such morphologies.

Wake and Larson [20] suggested that in the case of the plethodontid premaxilla, "an ancestral epigenetic default state [the bipartite state] can be produced whenever the system that generates the derived unipartite condition is perturbed". More specific to foot morphology, Shubin, et al. [31] examined variation in salamander carpal and tarsal arrangements and concluded that the diversity seen within and among taxa is the result of developmental interactions in the formation of mesopodal elements. These interactions appear to explain aspects of the aberrant *C*. *priscus* foot noted here.

Much of the variation seen in salamander tarsal arrangements occurs in the central area of the foot where the proximodistal and anteropoterior axes of chondrogenesis converge [31]. One common result is the generation of extra elements, and this is seen in the aberrant *C*. *priscus* foot seen here (eleven tarsals instead of nine). Another result is various patterns of element fusion. Of primary interest here is element “m” as first defined by Schmalhausen [51].

Element “m” is a small tarsal element sometimes seen in the central region of the mesopodium in salamanders but is more commonly fused with distal tarsal 4. In *Chiropterotriton* and *Aneides*, it is likely that “m” fuses instead with distal tarsal 5, thus resulting in the tarsal arrangement characteristic of these general and hypothesized as adaptive for arboriality. In this specimen of *C*. *priscus*, it appears as though the developmental process has resulted in a reversion to the ancestral condition in which element “m” fuses with distal tarsal 4 instead of 5.

Variants such as this “look backward” in a phylogentic sense, revealing an ancestral condition and suggesting that underlying developmental systems can explain not only such atavisms but can also help us to understand how changes in such systems can explain the evolution of “future” morphologies [31]. So, considered by itself, this single aberrant *C*. *priscus* foot, is an atavism that is homoplastic (in terms of distal tarsals 4 and 5) in relation to most other plethodontid genera, but is likely produced by a homologous developmental process. In this light, the homoplastic body shape of *C*. *priscus* might itself be the result of a developmental pathway retained by *C*. *priscus* but modified in all other species of the genus.

Although morphological homoplasy as a pattern in *Chiropterotriton* and plethodontid salamanders in general seems at first to strike us as roadblock to understanding evolutionary history, the homologous developmental systems that appear to underlie such homoplasy may reveal common and consistent evolutionary processes at work. Whatever the reason(s) for this repetition—conserved genes, common developmental systems, functional constraint—it seems to be further evidence that “it is easier for some things to evolve than for others” [6].

## Supporting Information

S1 Appendix Specimens used for osteological analysis. All specimens are from the permanent collections of the Museum of Vertebrate Zoology, University of California, Berkeley.
(DOCX)

S1 Permission Permission for the use of photos in Fig 1. (DOCX)
