# Supplementary material for: Osteological Variation among Extreme Morphological Forms in the Mexican Salamander Genus Chiropterotriton (Amphibia: Plethodontidae): Morphological Evolution And Homoplasy
Source: PLoS One. 2015 Jun 10;10(6):e0127248. doi: 10.1371/journal.pone.0127248 (PMC4464517; doi:10.1371/journal.pone.0127248)
Supplement: S1 Appendix — All specimens are from the permanent collections of the Museum of Vertebrate Zoology, University of California, Berkeley. (DOCX) [file pone.0127248.s001.docx]

**Individual MVZ* numbers of the specimens used for this study**

***Museum of Vertebrate Zoology, University of California, Berkeley**

*Chiropterotrition magnipes*

128249, 128258, 129010,129011, 129013, 129015, 129021, 129036, 129042, 129044

*Chiropterotrion priscus*

138871, 138883, 163887, 163888, 163890, 163892, 163902, 163976, 163977, 192794

*Chiropterotrition lavae*

106532, 106538, 106542, 106546, 163916, 171875, 171878, 171880, 171882, 171898

*Chiropterotrition dimidiatus*

103967, 106502, 106511, 114249, 114258, 114263, 114272, 114494, 118734, 185965
