# Supplementary material for: Osteological Variation among Extreme Morphological Forms in the Mexican Salamander Genus Chiropterotriton (Amphibia: Plethodontidae): Morphological Evolution And Homoplasy
Source: PLoS One. 2015 Jun 10;10(6):e0127248. doi: 10.1371/journal.pone.0127248 (PMC4464517; doi:10.1371/journal.pone.0127248)
Supplement: S1 Permission — (DOCX) [file pone.0127248.s002.docx]

**From:** Sean Rovito <[smrovito@gmail.com](mailto:smrovito@gmail.com)>

**Date:** Sunday, February 8, 2015 at 10:17 PM

**To:** Dave Darda <[dardad@cwu.edu](mailto:dardad@cwu.edu)>

**Subject:** Re: Chirop photos

Hi David,

Congats on getting the paper accepted - I look forward to seeing it in print. Do I need to email someone at PLoS One to give my permission, or is an email sufficient? In case this email counts:

I give permission for the open-access journal PLoS One to publish Figure 1 under the Creative Commons Attribution License (CCAL) CC BY 3.0 (<http://creativecommons.org/licenses/by/3.0/us/>).

Please let me know if I need to do something else or contact someone in order to give my permission to publish this figure.

Best,

Sean

On Feb 8, 2015, at 4:47 PM, David Darda <[DardaD@cwu.edu](mailto:DardaD@cwu.edu)> wrote:

Sean,

Good news!  The Chiropterotriton paper Dave Wake and I were working on has been accepted by PLOS ONE.  (Title:  Osteological variation among extreme morphological forms in the Mexican salamander genus Chiropterotriton (Amphibia: Plethodontidae): morphological evolution and homoplasy)

One of the requirements from the journal is to obtain your written permission as the original copyright holder of the images we are using in Figure 1 (attached).  The specific request wording given to us is:

“I request permission for the open-access journal PLOS ONE to publish Figure 1 under the Creative Commons Attribution License (CCAL) CC BY 3.0 (<http://creativecommons.org/licenses/by/3.0/us/>).  Please be aware that this license allows unrestricted use and distribution, even commercially, by third parties.  Please reply and provide explicit written permission to publish Figure 1 under a CC BY license.”

Let me know if you have any questions or concerns about this.  I look forward to hearing back.

Thanks again for your help and the great photos!

Dave

Dr. David Darda

Professor, Department of Biological Sciences

Central Washington University

Ellensburg, WA  98926

[dardad@cwu.edu](mailto:dardad@cwu.edu)

509-963-2881
